# The Duality of the MAPK Signaling Pathway in the Control of Metabolic Processes and Cellulase Production in *Trichoderma reesei*

**DOI:** 10.1038/s41598-018-33383-1

**Published:** 2018-10-08

**Authors:** Renato Graciano de Paula, Amanda Cristina Campos Antoniêto, Cláudia Batista Carraro, Douglas Christian Borges Lopes, Gabriela Felix Persinoti, Nalu Teixeira Aguiar Peres, Nilce Maria Martinez-Rossi, Rafael Silva-Rocha, Roberto Nascimento Silva

**Affiliations:** 1 Molecular Biotechnology Laboratory, Department of Biochemistry and Immunology, Ribeirao Preto Medical School (FMRP), University of Sao Paulo, Ribeirao Preto, SP Brazil; 2 Laboratório Nacional de Ciência e Tecnologia do Bioetanol (CTBE), Centro Nacional de Pesquisa em Energia e Materiais (CNPEM), Campinas, SP Brazil; 3 Center of Biological Science and Health (CCBS), Sergipe Federal University (UFS), Aracaju, SE Brazil; 4 Department of Genetics, Ribeirão Preto Medical School, University of São Paulo, Ribeirão Preto, SP Brazil; 5 Systems and Synthetic Biology Laboratory, Department of Cell and Molecular Biology, Ribeirao Preto Medical School (FMRP), University of Sao Paulo, Ribeirao Preto, SP Brazil

## Abstract

In this study, through global transcriptional analysis by RNA-Sequencing, we identified the main changes in gene expression that occurred in two functional mutants of the MAPK genes *tmk1* and *tmk2* in *Trichoderma reesei* during sugarcane bagasse degradation. We found that the proteins encoded by these genes regulated independent processes, sometimes in a cross-talk manner, to modulate gene expression in *T. reesei*. In the Δ*tmk2* strain, growth in sugarcane bagasse modulated the expression of genes involved in carbohydrate metabolism, cell growth and development, and G-protein-coupled receptor-mediated cell signaling. On the other hand, deletion of *tmk1* led to decreased expression of the major genes for cellulases and xylanases. Furthermore, TMK1 found to be involved in the regulation of the expression of major facilitator superfamily transporters. Our results revealed that the MAPK signaling pathway in *T. reesei* regulates many important processes that allow the fungus to recognize, transport, and metabolize different carbon sources during plant cell wall degradation.

## Introduction

The filamentous fungus *Trichoderma reesei* is one of the major sources for industrial cellulases production^1^. *T. reesei* has a small repertoire of cellulases and hemicellulases, the smallest among biomass-degrading fungi. However, its cellulolytic enzyme system is the most efficient among filamentous fungi^2^. Cellulase expression is mainly regulated by the transcription factors XYR1, ACE2, and the Heme Activator Protein complex (HAP2/3/5) as positive regulators, and CRE1 and ACE1 as negative regulators^3,4^. Recently, other functional cellulase regulators, such as LAE1, VELVET, and ACE3, have been described^5–8^. However, it remains unclear how exactly the induction signals are delivered. Several signal transduction mechanisms and factors have been reported to influence cellulase expression in *T. reesei*. Among them, GNA1 and GNA3, two G-alpha subunits, respond to different carbon sources and are involved in regulating cellulolytic enzymes^9,10^. Additionally, the influence of cAMP levels as well as signal transduction involving light on cellulase gene expression has been discussed^11–14^.

The MAPK signaling pathway is an evolutionarily conserved cascade involved in transducing extracellular signals, induced by adequate cellular responses^15–17^. In animals, plants, and fungi, MAPK signaling regulates transcription factor function through MAPK-mediated phosphorylation. In fungi, the MAPK signaling pathway has been well studied in *Saccharomyces cerevisiae*^18^, and it controls a range of processes, such as fruiting body development, polarized growth, pathogenicity, circadian rhythm, conidiation, protein biosynthesis and secretion, stress response, and cell wall integrity^19–25^. In filamentous fungi, three *S. cerevisiae* homologous MAPKs, *Hog1*, *Slt2*, and *Fus3*, have been identified^24,26,27^. In the *T. reesei* database (http://genome.jgi-psf.org/Trire2/Trire2.home.html), three genes are annotated to encode MAPK pathway members (*tmk1, tmk2, and tmk3)*. Recent reports have shown that *tmk2, tmk1*, and *tmk3* signaling pathways may control different processes in this fungus, including cell wall integrity, cellulase production, biomass accumulation, sporulation, and resistance to high osmolarity^24,28–30^, however, these studies only examined the expression of a small number of genes. In the current study, we conducted a RNA sequencing-based global gene expression analysis to reveal the functions of TMK1 and TMK2 in *T. reesei*. In addition, our study provides the most complete insight to date about the function of TMK1 and TMK2 in *T. reesei* and it contributes to the understanding of signal transduction processes that control cellulases production in this important industrial fungus.

## Results

### *tmk1* and *tmk2* MAPK Genes Are Induced in *T. reesei* in the Presence of Sugarcane Bagasse

**Figure 1:**
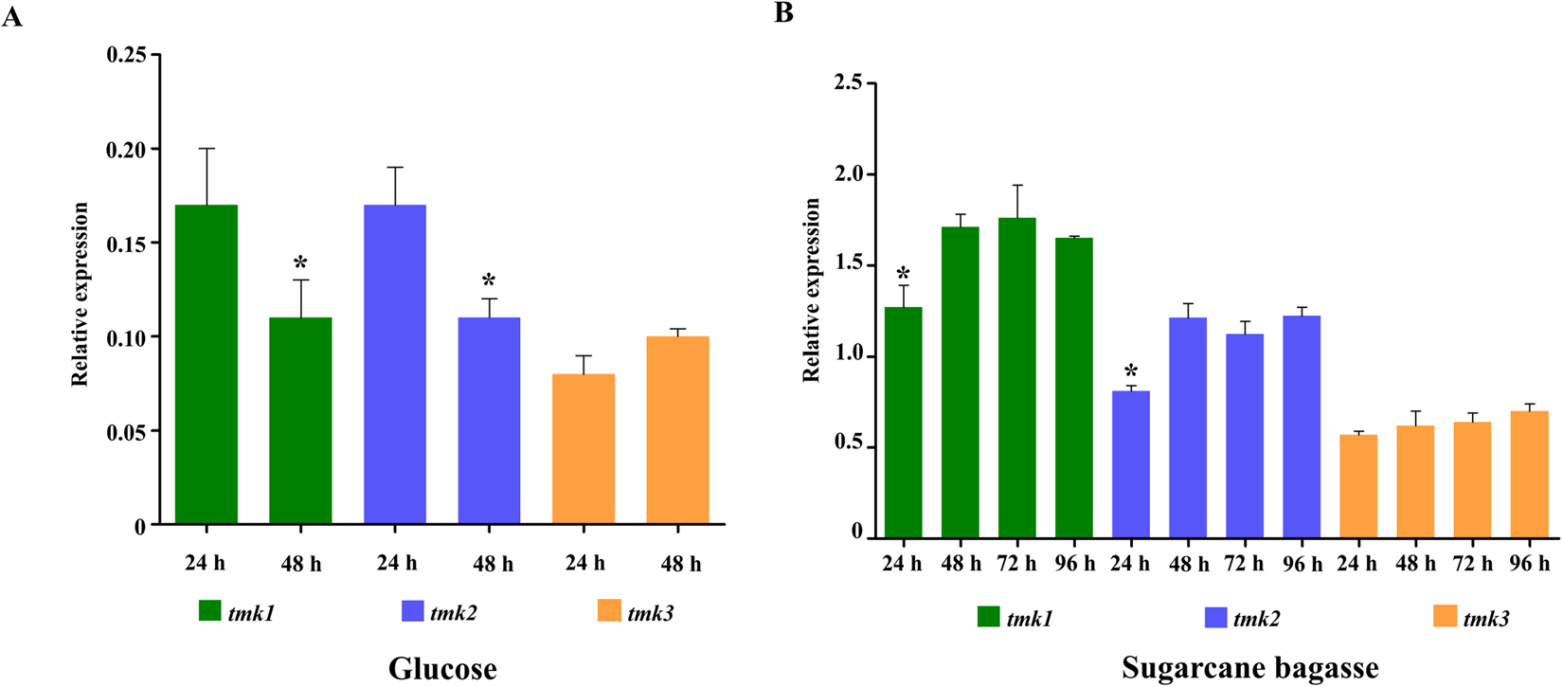
Expression profile of MAPK genes in the QM6a parental strain grown in sugarcane bagasse and glucose. (**A**) Relative expression of *tmk1, tmk2*, and *tmk3* upon growth in glucose. Expression levels were calibrated according to the comparative 2^−ΔCt^ method, using the constitutively expressed gene β-actin as an endogenous control (ANOVA followed by Tukey’s pairwise comparison P < 0.05). *Significantly different from 24 h (P < 0.05). (**B**) Relative expression of *tmk1, tmk2*, and *tmk3* in the presence of sugarcane bagasse. Expression levels were calibrated according to the comparative 2^−ΔΔCt^ method, using the constitutively expressed gene β-actin as an endogenous control and glycerol samples the reference group (ANOVA followed by Tukey’s pairwise comparison P < 0.0001). *Significantly different from 48 h, 72 h, and 96 h (P < 0.0001). These results are based on three replicates of three independent experiments and are expressed as mean ± standard deviation.

*In silico* analysis of the *T. reesei* genome (http://genome.jgi-psf.org/Trire2/Trire2.home.html) showed that this fungus has 3 genes (*tmk1, tmk2*, and *tmk3*) encoding MAPK signaling pathway members. Additionally, several reports have suggested the involvement of this pathway in regulating cellulase expression^24,28,30^. Thus, we investigated the involvement of these MAPK genes in the regulation of cellulase expression in *T. reesei* during sugarcane bagasse degradation. First, we profiled MAPK genes expression in the QM6a parental strain grown in the presence of sugarcane bagasse, an inductive carbon source, or glucose, which is a repressive carbon source for cellulase synthesis. As demonstrated in Fig. 1A, the MAPK genes were expressed at low levels in the presence of glucose, with a slight increase at 24 hours for *tmk1* and *tmk2*, and at 48 hours for *tmk3*. In sugarcane bagasse, MAPK genes expression was analyzed after 24, 48, 72, and 96 hours of cultivation (Fig. 1B). The *tmk1* and *tmk2* genes showed the highest expression from 48 hours to 96 hours in the presence of sugarcane bagasse, and these levels were significantly higher than the levels observed at 24 hours. No changes in *tmk3* expression were observed over time in the presence of sugarcane bagasse (Fig. 1B). Therefore, we selected *tmk1* and *tmk2* for functional studies. *T. reesei* Δ*tmk2* and Δ*tmk1* strains were constructed by homologous recombination using QM6aΔ*tmus53*Δ*pyr4*, in which the non-homologous end joining pathway is disrupted^31^, as the parental strain (Additional file 1).

### Deletion of *tmk2* Impairs Growth in Presence of Lactose and Glucose

Phenotypic analysis was conducted for parental, *Δtmk1*, and *Δtmk2* strains by growing these strains on agar plates with different medium compositions (Supplementary Fig. 3A,D). The *Δtmk1* strain showed no difference in growth, sporulation, and biomass formation compared to the parental strain (Supplementary Fig. 3B,C). The *Δtmk2* did not differ from the parental strain in sporulation patterns either. However, reduction of growth was observed for the *Δtmk2* mutant strain when grown in minimal medium supplemented with lactose and glucose (Supplementary Fig. 3D). In addition, we observed that *Δtmk2* showed difference in growth profiles (Supplementary Fig. 3E) and similar biomass accumulation after growth on glycerol for 24 hours (Supplementary Fig. 3E).

### Gene Regulatory Network Controlled by *tmk1* and *tmk2* in *T. reesei*

*T. reesei* QM6a, Δ*tmk1*, and *Δtmk2* strains were cultivated in presence of sugarcane bagasse, glycerol, and glucose as carbon sources, and three biological replicates of each condition were submitted for RNA-Sequencing using an Illumina HiSeq™ 2500 (Additional file 3).

**Figure 2:**
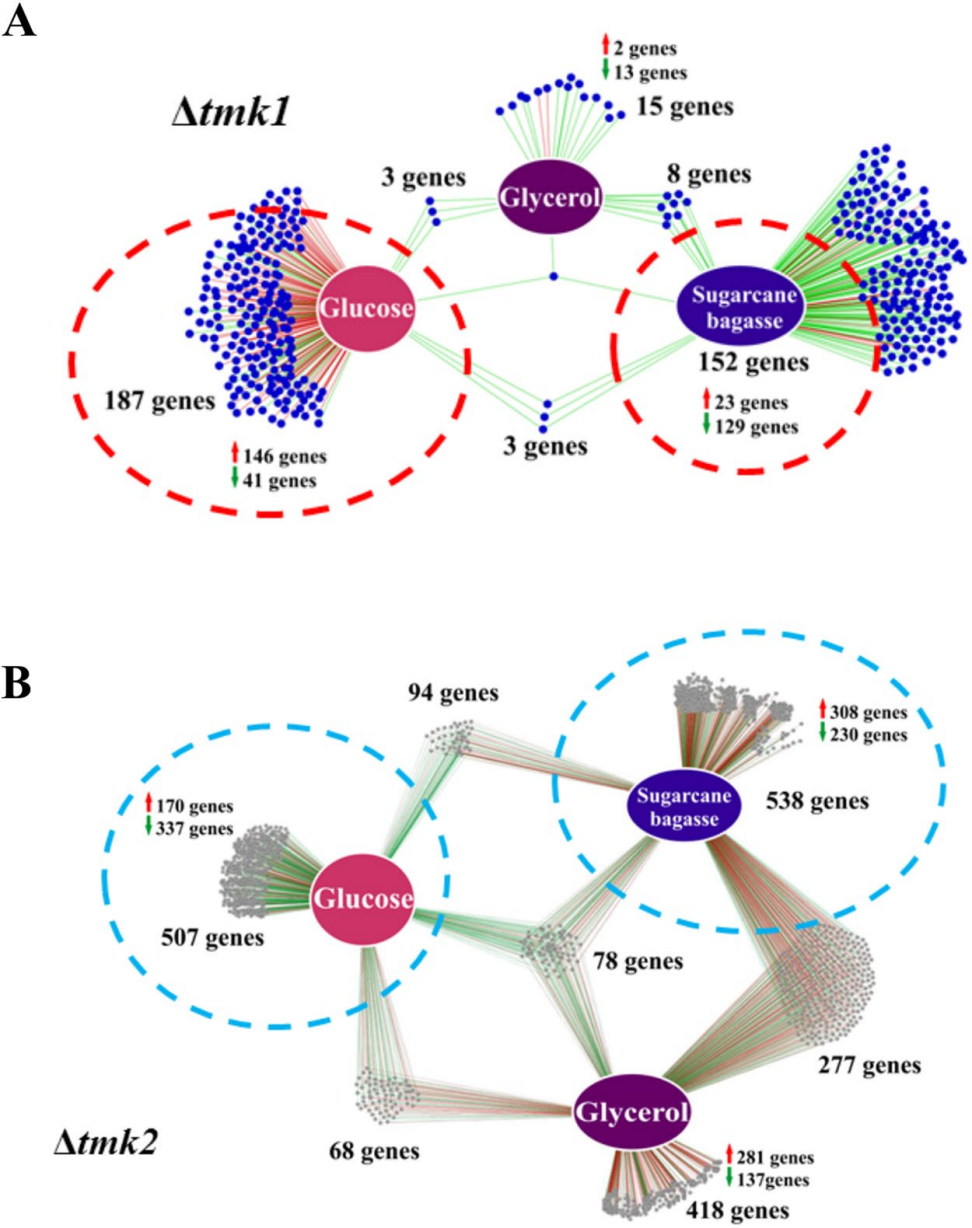
Gene Regulatory Network (GRN) of differentially expressed genes in the mutant strains grown in glucose, sugarcane, and glycerol. (**A**) GRN of 402 differentially expressed genes in the Δ*tmk1* compared to the QM6a (Δ*tmk1/*QM6a) in the presence of glucose, glycerol, and sugarcane bagasse. Genes are represented as nodes (circles), and interactions are represented as edges (red lines: upregulated interactions, green lines: downregulated interactions), connecting the nodes: 382 interactions, (**B**) GRN of 2575 differentially expressed genes in the Δ*tmk2* compared to the QM6a (Δ*tmk2/*QM6a) in the presence of glucose, glycerol, and sugarcane bagasse. Genes are represented as nodes (shown as circles), and interactions are represented as edges (red lines: upregulated interactions, green lines: downregulated interactions), connecting the nodes: 1980 interactions.

From the RNA-Seq data, regulatory networks were built to identify genes that were specifically expressed in a determined growth condition (Fig. 2). In the Δ*tmk1* regulatory network (Fig. 2A), 152 genes were exclusively regulated during growth in the presence of sugarcane bagasse (23 up- and 129 downregulated), 187 exclusively in the presence of glucose (146 up- and 41 downregulated), and 15 exclusively in the presence of glycerol (two up- and 13 downregulated) (Supplementary Table 2), while only 1 gene was commonly regulated among the three conditions examined, when compared to the parental strain. Moreover, three genes were common regulated between the sugarcane bagasse and glucose conditions, three between the glucose and glycerol conditions, and eight between the sugarcane bagasse and glycerol conditions, when compared to the parental strain (Fig. 2A). In the Δ*tmk2* mutant strain, compared to QM6a, 538 genes were exclusively regulated during growth in sugarcane bagasse (308 up- and 230 downregulated), 507 exclusively in glucose (170 up- and 337 downregulated), and 418 exclusively in glycerol (281 up- and 137 downregulated) (Supplementary Table 3), while 78 genes were commonly regulated among the three conditions examined (Fig. 2B). Moreover, 94 genes were common regulated between the sugarcane bagasse and glucose conditions, 68 between the glucose and glycerol conditions, and 277 between the sugarcane bagasse and glycerol conditions (Fig. 2B). Genes differentially expressed in Δ*tmk1* and Δ*tmk2* compared to QM6a in the presence of sugarcane bagasse were functionally annotated using Gene Ontology (GO) terms (Supplementary Fig. 9A). The main gene categories included integral to membrane, carbohydrate transport, L-arabinose isomerase activity, sugar:proton symporter activity, and DNA binding activity (Supplementary Fig. 9A). On the other hand, genes involved in transport, nucleic acid binding, hydrolase activity, RNA binding, synthase activity, copper ion transport, chromatin assembly and disassembly and chromatin were the GO terms most enriched in the Δ*tmk2* mutant strain grown in sugarcane bagasse when compared to the parental strain (Supplementary Fig. 9B). We selected the 90 genes that showed the strongest up- or downregulation (Log2 fold change ≥1 or ≤−1 and adjusted p ≤ 0.05) during growth of Δ*tmk1* and Δ*tmk2* in the different carbon sources analyzed (Supplementary Tables 4 and 5). In Δ*tmk1*, when compared to QM6a, the upregulated genes during growth in sugarcane bagasse were mainly related to aromatic compound metabolism, oxidoreductase activity, and the membrane. The most strongly expressed gene encoded FAD-binding domain-containing protein (ID 62463) (13-fold), followed by a gene encoding an unknown protein (ID 105447) (6.5-fold), and one encoding a short chain dehydrogenase/reductase (ID 70520) (6-fold). Two genes encoding major facilitator superfamily (MFS) permeases (ID 65915 and ID 57749) and two genes encoding transcription factors (ID 111446 and ID 54395) were also among the most strongly upregulated genes in Δ*tmk1* in sugarcane bagasse when compared to the parental strain (Supplementary Table 4). On the other hand, the most strongly downregulated genes in Δ*tmk1* in presence of sugarcane bagasse when comparing the mutant to the parental strain were mainly related to carbohydrate metabolism and cellulase activity. The most strongly downregulated gene encoded a MFS permease (ID 54005) (70-fold), followed by two xylanase encoding genes (ID 120229 and ID 123818) (34.5 and 28-fold, respectively) and one encoding an unknown protein (ID 39587) (34.5-fold) (Supplementary Table 4).

In Δ*tmk2*, when compared to QM6a, the most strongly upregulated genes in the presence of sugarcane bagasse encoded a β-lactamase class C (ID 58717) (75-fold), an MFS permease (maltose permease) (ID 65191) (38-fold), and an amidase (ID 78072) (36-fold). Interestingly, the expression of two glycosyl hydrolase-encoding genes (GH105/GH88 glycosyl hydrolase, ID 57179; and GH11 endo-β-1,4-xylanase XYN5, ID 112392) were increased at least 24- and 23-fold in the mutant relative to the parental strain, respectively (Supplementary Table 5). Regarding the downregulated genes in the three carbon sources, 62% were of unknown function. In the presence of sugarcane bagasse, the most strongly downregulated gene (ID 108676) encoded an unknown protein secreted only by Ascomycota, which exhibited a >161-fold decrease in Δ*tmk2* compared to QM6a. The second-most repressed gene in Δ*tmk2* upon growth in sugarcane bagasse encoded a transporter belonging to the MFS permease family (ID 103179), showing >117-fold decrease in expression compared to the parental strain (Supplementary Table 5).

**Figure 3:**
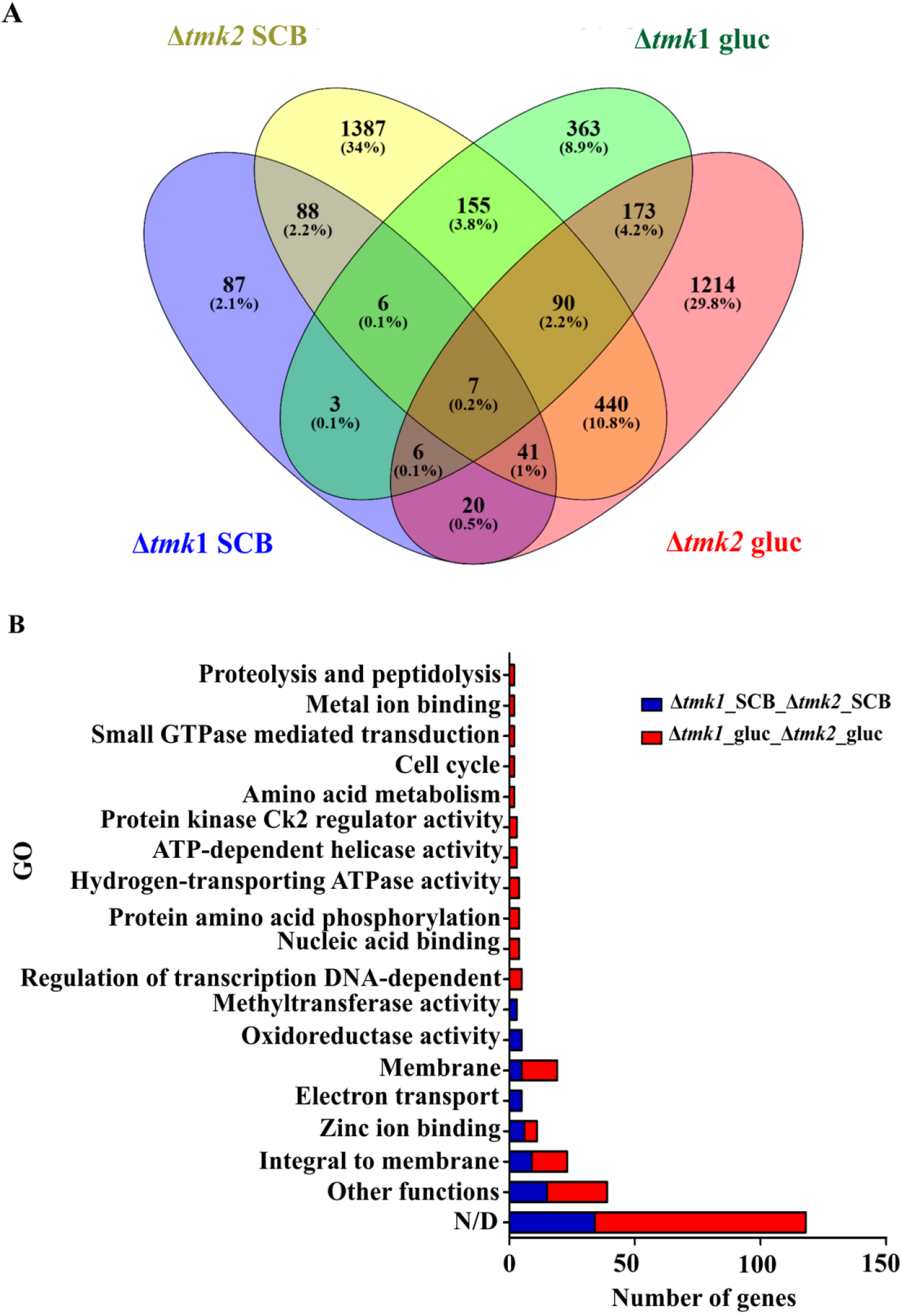
Expression pattern of genes commonly expressed between the Δ*tmk1* and Δ*tmk2* representing the number of differentially expressed genes in the presence of glucose and sugarcane bagasse. (**A**) Comparative Venn diagram of commonly expressed genes between the Δ*tmk1* and Δ*tmk2* strains in the presence of glucose and sugarcane bagasse. Venn diagram clustering was designed using Venny 2.1 tools. (**B**) Gene Ontology (GO) enrichment analysis of commonly expressed genes between the Δ*tmk1* and Δ*tmk2* strains in the presence of glucose and sugarcane bagasse. The enriched GO terms according to molecular, cellular component, and biological process in *T. reesei*. Significantly enriched categories (P ≤ 0.05) are shown. The threshold for calling differentially expressed genes was P ≤ 0.05.

To identify potential cross- talk between the TMK1 and TMK2 signaling in the presence of sugarcane bagasse and glucose, we selected the genes whose expression was differentially regulated in both mutant strains when compared to the parental strain. As seen in Fig. 3A, 88 genes were regulated in both Δ*tmk1* and Δ*tmk2* in the presence of sugarcane bagasse (approximately 86% of genes were commonly up- or downregulated) (Supplementary Table 6), while 173 genes were regulated in the mutant strains in the presence of glucose in relation to the parental strain (approximately 41% of genes were commonly up- or downregulated) (Supplementary Table 7). GO enrichment analysis (Fig. 3B) showed that the commonly regulated genes in the mutant strains in the presence of sugarcane bagasse mainly encoded proteins with no described function; followed by GO terms related to integral to membrane, zinc ion binding, electron transport, membrane, and oxidoreductase activity.

### TMK1 and TMK2 are Involved in the Regulation Hollocellulase Expression and Activity

**Figure 4:**
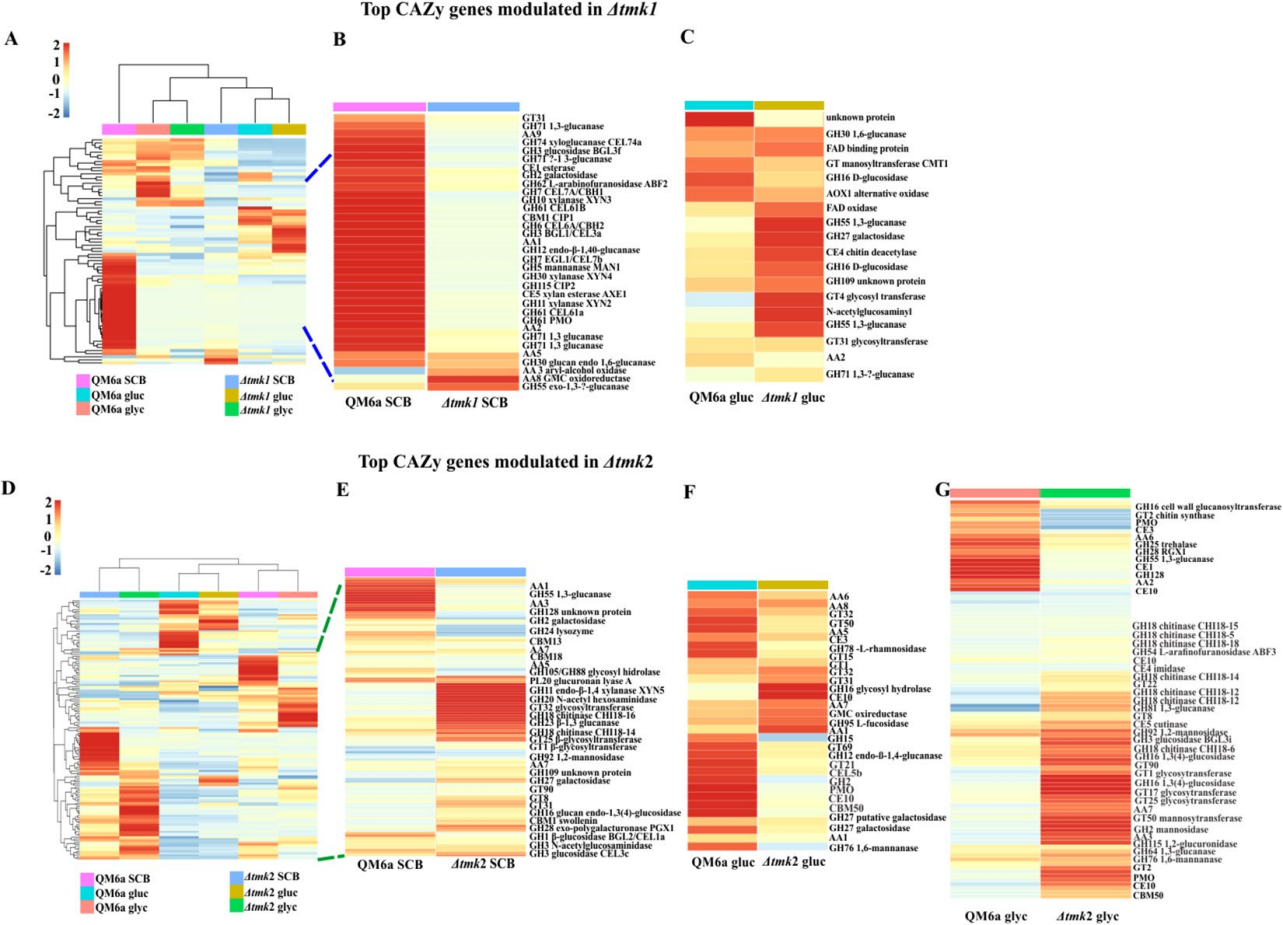
Cellulase gene expression profile of *T. reesei* QM6a, Δ*tmk1*, and Δ*tmk2* strains during growth in glucose (gluc), glycerol (glyc), and sugarcane bagasse (SCB). (**A**) Heatmap of differentially expressed CAZy genes in the Δ*tmk1* and QM6a strains showing all the conditions of this study. (**B**) Heatmap of differentially expressed CAZys in Δ*tmk1* and QM6a strains grown in sugarcane bagasse. (**C**) Heatmap of differentially expressed CAZys of Δ*tmk1* and QM6a strains grown in glucose. (**D**) Heatmap of differentially CAZy genes in the Δ*tmk2* and QM6a strains showing all the conditions of this study. (**E**) Heatmap of differentially expressed CAZys in Δ*tmk2* and QM6a strains grown in sugarcane bagasse. (**F**) Heatmap of differentially expressed CAZys from Δ*tmk2* and QM6a strains grown in glucose. (**G**) Heatmap of differentially expressed CAZys from Δ*tmk2* and QM6a strains grown in glycerol. The hierarchical clustering was performed using the R *pheatmap* package. Complete linkage method and Euclidean distance of row centered and scaled TPM values were used for hierarchical clustering of the differentially expressed genes in all analyzed conditions.

An overview of the possible roles for TMK1 and TMK2 in cellulase gene expression is shown in Fig. 4. In the presence of sugarcane bagasse or glucose, CAZyme-encoding genes were significantly up- and downregulated in Δ*tmk1* when compared to QM6a (Fig. 4B,C and Supplementary Table 13). In the presence of sugarcane bagasse, the deletion of *tmk1* negatively affected the expression of the major hollocellulases produced by *T. reesei*. Only one gene (encoding an oxidoreductase family protein) was upregulated in Δ*tmk1* in this carbon source as compared to the parental strain (Supplementary Table 13).

The major downregulated holocellulase genes in Δ*tmk1* in the presence of sugarcane bagasse encoded xylanases XYN3 (ID 120229) (35-fold), XYN2 (ID 123818) (29-fold), and XYN4 (ID 111849) (7-fold) (Fig. 4B). Five genes related to xylan metabolism, encoding CE5 acetyl xylan esterase AXE1 (ID 73632), CE5 acetyl xylan esterase (ID 54219), GH74 xyloglucanase CEL74a (ID 49081), and the L-arabinofuranosidases ABF2 (ID 76210) and ABF3 (55319) were also downregulated in Δ*tmk1* in this condition (Fig. 4B and Supplementary Table 13). In addition, the two major cellulase genes of *T. reesei*, *cel7a* (CBH1; ID 123989), and *cel6a* (CBH2; ID 72567) also exhibited decreased expression in the presence of sugarcane bagasse (Log2 fold change = −3.93 and −3.69, respectively) (Fig. 4B and Supplementary Table 13). The expression of β-glucosidases and endoglucanases was also altered upon *tmk1* deletion in this condition. The genes *cel3a* (BGL1; ID 76672), *cel1a* (BGL2; ID 120749), *cel5a* (EGL2; ID 120312), and *cel7b* (EGL1; ID 122081) exhibited 19-, 7-, 16-, and 14-fold decreased expression, respectively, in Δ*tmk1* compared to QM6a grown in sugarcane bagasse (Fig. 4B). Furthermore, three genes coding for lytic polysaccharide monooxygenases (LPMOs) and four genes coding for auxiliary activities (AA1 family) were also downregulated in Δ*tmk1* in this condition (Fig. 4B and Supplementary Table 13).

In Δ*tmk2*, a different cellulase gene expression profile was observed. In the three carbon sources analyzed, CAZyme-encoding genes were up- or downregulated in Δ*tmk2* when compared to QM6a (Fig. 4D–G). Notably, in the presence of glucose and glycerol, genes related to cell wall remodeling, such as chitinases, mannanase, galactosidase, glycosyl transferase, and glucanases, were upregulated when compared to the parental strain (Fig. 4F,G). In this way, TMK2 can act both as a positive or negative regulator of cellulase gene expression and it seems to control basal development of *T. reesei*.

**Figure 5:**
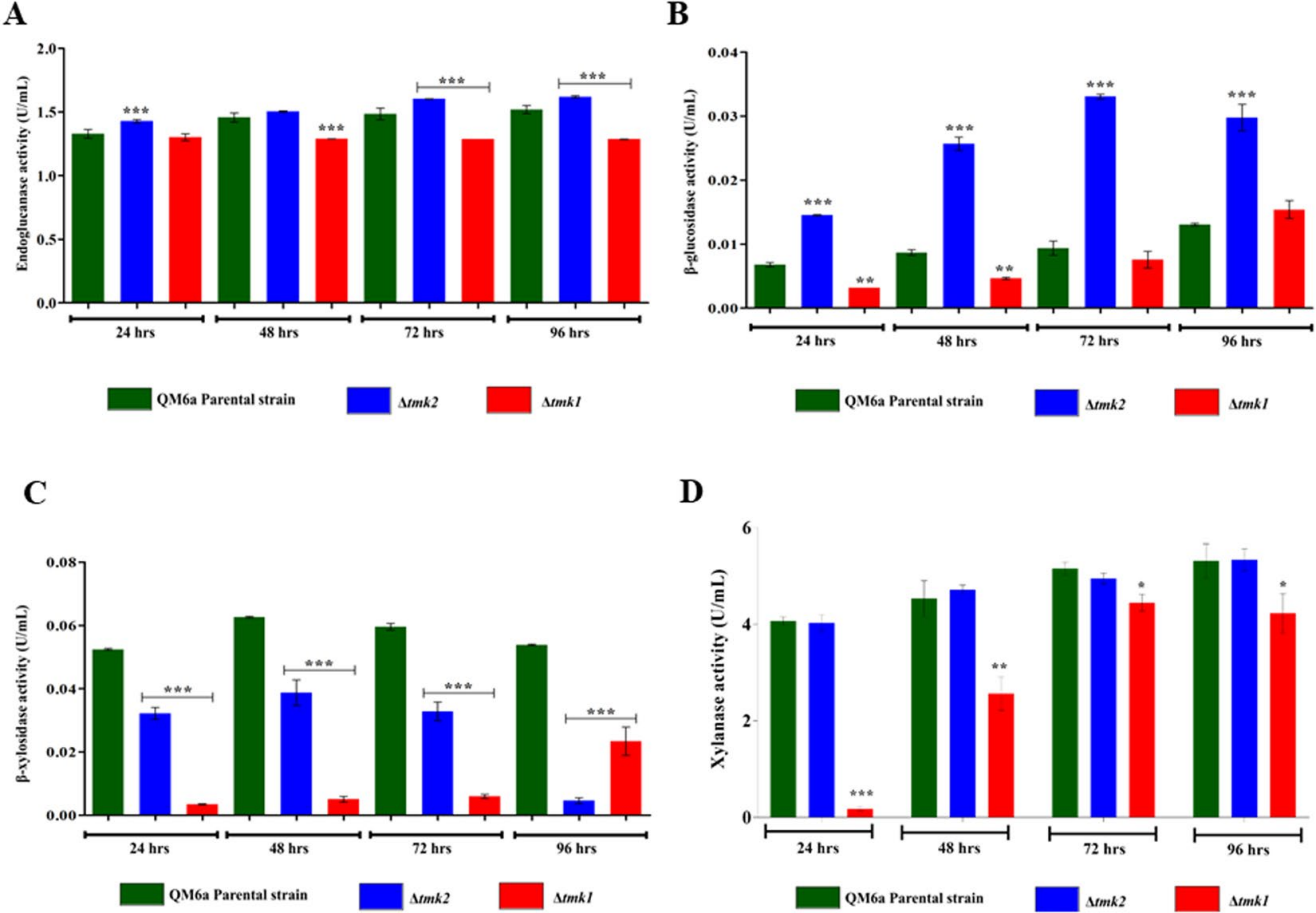
Holocellulolytic activities of QM6a, Δ*tmk2*, and Δ*tmk1* grown in sugarcane bagasse. (**A**) Endoglucanase activity (CMCase), (**B**) β-glucosidase, (**C**) β-xylosidase and (**D**) Xylanase activities from culture supernatant of *T. reesei*, QM6a parental strain, Δ*tmk2*, and Δ*tmk1* grown in the presence of sugarcane bagasse for the indicated times. ***Significantly different from the QM6a parental strain (P < 0.001) and *Significantly different from the QM6a parental strain (P < 0.05).

Enzymatic activity analysis was performed to determine whether TMK1 and TMK2 affect the activity of holocellulolytic enzymes in *T. reesei* during sugarcane bagasse degradation. The endoglucanase (CMCase), β-glucosidase, β-xylosidase and xylanases activities were profiled in *Δtmk2, Δtmk1*, and QM6a (Fig. 5). Regarding the specific CMCase activity, QM6a and *Δtmk2* presented similar profiles, with slightly increased activity in *Δtmk2* at 24, 72, and 96 hours. On the other hand, CMCase activity in Δ*tmk1* decreased over cultivation (Fig. 5A). β-glucosidase activity was higher in *Δtmk2* at 24, 48, 72, and 96 hours than in the parental strain and *Δtmk1* (Fig. 5B). In the same assay, *Δtmk1* presented significantly lower β-glucosidase activity than QM6a at 24 and 48 hours. Both mutants showed significantly lower β-xylosidase activity than the QM6a parental strain, but with different patterns (Fig. 5C). The *Δtmk2* mutant presented reduced activity at 72, and 96 hours in comparison to the parental strain. Furthermore, *Δtmk1* strain showed reduced β-xylosidase activity over time, when compared to the QM6a strain (Fig. 5C). Regarding the specific xylanase activity, QM6a and *Δtmk2* presented similar profiles (Fig. 5D). In the same assay, *Δtmk1* presented significantly lower xylanase activity than QM6a at 24, 48, 72 and 96 hours (Fig. 5D). Thus, the MAPKs TMK1 and TMK2 might affect the activity of holocellulolytic enzymes in *T. reesei* upon exposure to sugarcane bagasse, being this effect due to the regulation of cellulase genes in the mutant strains.

### TMK2 Regulates Crosstalk Between Different Signaling Pathways in *T. reesei*

The RNA-Seq data demonstrated that the expression of distinct genes belonging to various signaling pathways was altered upon *tmk2* deletion (Supplementary Table 8). In the presence of sugarcane bagasse, genes encoding G protein–coupled receptors (GPCRs); phospholipases C, A2, and D; kinase proteins; Ras-GTPase; calcium and histidine kinase; calmodulin; calcipressin; GNA1; casein kinase 1; and MAPKKK (Ste11) were differentially expressed in Δ*tmk2* (Supplementary Table 8). Interestingly, the most strongly differentially regulated genes from different pathways were involved in growth and development, reinforcing the role of TMK2 in the control of basal development of *T. reesei*. Among the genes upregulated in Δ*tmk2* in the presence of sugarcane bagasse when compared to the parental strain, we can highlight a gene-encoding conidiospore surface protein 1 (CMP1) (ID 72379) (13.7-fold); a gene (ID 64167) encoding a protein involved in sexual differentiation (10-fold); and three genes encoding GPCR PTH11 (an integral membrane protein required for pathogenicity) isoforms (IDs 109146, 122824, and 27992). These genes were 5-, 3-, and 2-fold increased, respectively (Supplementary Table 8). Additionally, genes encoding other GPCRs (IDs 123806 and 63981) were upregulated in Δ*tmk2* (4- and 3-fold, respectively) compared to QM6a. Finally, genes encoding a cell cycle protein (ID 64125, 5.8-fold), phospholipase C (ID 21960, 3-fold), and phospholipase D (ID 22331, 2.7-fold) were the most strongly expressed genes in Δ*tmk2* (Supplementary Table 8) in the same condition.

Among the downregulated genes, GPCR-coding genes were overrepresented (Supplementary Table 8). Eight genes encoding GPCRs showed 1.6–5.25-fold decreases in expression in Δ*tmk2* in comparison to the QM6a. Moreover, a gene encoding a serine-threonine protein kinase (ID 112669) was repressed at almost 6-fold in the mutant strain in the presence of sugarcane bagasse. Interestingly, our results demonstrated that genes related to calcium signaling were also repressed in Δ*tmk2* grown in sugarcane bagasse (Supplementary Table 8). In addition, the histidine kinase HHK6 gene and eight other genes encoding kinase proteins were nearly 1.2-fold repressed in this condition. Similarly, the expression of upstream components of MAPK signaling was altered by *tmk2* deletion. Genes encoding a mitogen-activated protein kinase kinase kinase (MAPKKK STE11; ID 4945) and its regulator MAPKKK STE50 (ID 105409), were 1.7-fold and 1.6-fold repressed, respectively, in the mutant strain in the presence of sugarcane bagasse (Supplementary Table 8), suggesting the TMK2-dependent regulation of MAPK signaling at various points.

**Figure 6:**
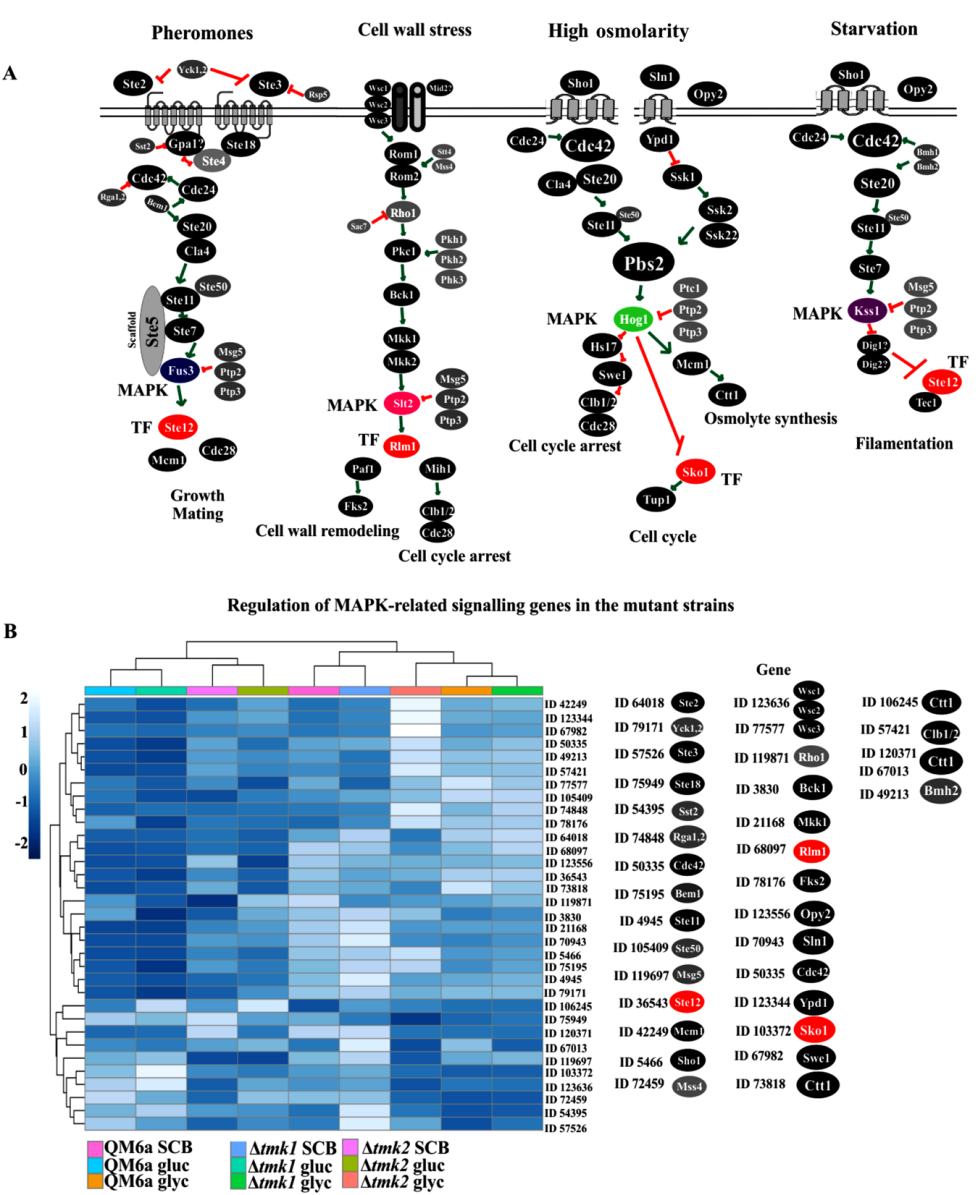
Global view of the MAPK signaling pathway in Δ*tmk2* and Δ*tmk1* mutant strains grown in glucose, glycerol, and sugarcane bagasse. (**A**) Functional reconstruction of the main MAPK signaling pathways involved in pheromone response, cell wall stress, high osmolarity, and starvation in *T. reesei*. This analysis was performed using the KEGG MAPK signaling pathway in Yeast (https://www.genome.jp/kegg/pathway/sce/sce04011.html) as reference. (**B**) Heatmap showing expression of the differentially expressed genes belonging to the MAPK signaling pathway in the *T. reesei* mutant strains. The hierarchical clustering was performed using the R *pheatmap* package. Complete linkage method and Euclidean distance of row centered and scaled TPM values were used for hierarchical clustering of the differentially expressed genes in all analyzed conditions. The gene IDs represent the genes differentially expressed in the mutant strains grown in glucose, glycerol, and sugarcane bagasse.

Using the KEGG MAPK signaling pathway for yeast (www.genome.jp/kegg/pathway/sce/sce04011.html) as a reference, we drafted the main MAPK signaling pathways in *T. reesei* (Fig. 6A). This global view revealed that transcriptional oscillations in genes encoding MAPK pathways occur in a carbon source-dependent manner (Fig. 6B). Regarding the pathway involved in pheromone response, a gene encoding a G protein-coupled receptor (ID 64018) was one of the most strongly repressed genes in Δ*tmk2*, exhibiting a 4-fold decrease in expression in the presence of glycerol. Next, a phosphatase enzyme-coding gene (ID 119697) showed a 2.5-fold decrease in sugarcane bagasse, 4.6-fold in glucose, and 2.3-fold in glycerol compared to the parental strain. Curiously, this gene is involved in both cell wall stress and the starvation response, which seems to be a crucial point of regulation controlled by TMK2. On the other hand, a gene encoding another G protein-coupled receptor (ID 57526) and the G-protein signaling regulator protein (ID 54395) were the most strongly induced in the presence of glycerol, showing 8-fold and 2-fold increases in expression in the mutant strain, respectively. Also, genes with IDs 67982 and 106245, involved in the high osmolarity response, showed 2-fold and 6-fold increased expression in the mutant strain in the presence of glycerol and sugarcane bagasse, respectively, in comparison to the parental strain. Regarding the downregulated genes, the genes encoding a transcription factor Sko1 and two isoforms of Ctt1 protein involved in high osmolarity response, (IDs 103372, 67013, and 73818, respectively) showed 2-fold, 3.3-fold, and 2.6-fold lower expression in Δ*tmk2* grown in glucose and glycerol (Fig. 6B). Finally, a gene encoding the transcription factor Tec1 (ID 108775) related to the starvation response was expressed 7.7-fold higher in Δ*tmk2* in response to glycerol. These results suggested that *tmk2* deletion might alter up- and downstream signaling through all MAPK pathways in *T. reesei*, impairing crucial processes in fungal development.

### TMK1 and TMK2 Modulate the Expression of Transcription Factors

To understand the transcriptional regulation of cellulases by TMK1 and TMK2, we analyzed the mRNA statuses of transcription factors in the mutant strains. The general expression profile of transcription factors was substantially altered in Δ*tmk1* and Δ*tmk2* (Supplementary Tables 9 and 10). Interestingly, none of the transcription factors known to directly regulate cellulase gene expression in *T. reesei* exhibited altered expression, suggesting an indirect relationship between MAPK and cellulase expression.

The changes in transcription factor gene expression profiles in the *Δtmk1* mutant compared to the parental strain were subtler than those observed in the *Δtmk2* mutant strain (Supplementary Table 9). In the presence of sugarcane bagasse, only six transcription factors were differentially expressed. The most strongly upregulated transcription factor, a Zn2Cys6 transcriptional regulator (ID 111446), was expressed 2.7-fold higher in Δ*tmk1* than in QM6a. FlbA (encoding a developmental regulator) (ID 54395) was the second most induced gene in *Δtmk1* grown in sugarcane bagasse (2.6-fold). In addition, two other genes encoding Zn2Cys6 transcriptional regulators (IDs 104182 and 79725) were equally upregulated in the presence of sugarcane bagasse, showing 2.11-fold and 2.03-fold increases. PacG/VIB-1 (ID 54675) was the only transcription factor downregulated (2.4-fold) in *Δtmk1* in the presence of sugarcane bagasse (Supplementary Table 9).

Thirty-four transcription factor-coding genes were modulated in Δ*tmk2* in the presence of sugarcane bagasse. Most encoded Zn2Cys6 transcriptional regulators (18 genes), C2H2 (6 genes), *bZIP* (3 genes), and *myb* (3 genes) families (Supplementary Table 10). The most strongly transcriptionally upregulated transcription factor in sugarcane bagasse in Δ*tmk2* compared to the parental strain (ID 105269, 7-fold) was a homolog of the Zn2Cys6 transcription regulator of *Aspergillus nidulans* AlcR, which is involved in ethanol regulon gene expression regulations. Furthermore, a homolog of the *myb* gene (ID 4124), previously described as a regulator of nitrogen metabolism in *A. nidulans*, was expressed 5.6-fold higher in the mutant strain than in QM6a. Curiously, another *myb* family member was one of the transcriptionally downregulated transcription factors upon growth in sugarcane bagasse (ID 1941, 2-fold) (Supplementary Table 10). This result suggests a refined dual mechanism that might control nitrogen metabolism in *T. reesei* by modulating transcription factors belonging to the *myb* family. Moreover, two genes encoding a bZIP transcription factor (ID 72524, 10-fold) and a C2H2 family member (ID 122448, 9.6-fold) were the most repressed genes upon growth in sugarcane bagasse (Supplementary Table 10). Altogether, these results suggest that *tmk1* and *tmk2* deletion changed the transcriptional response of transcription factors in *T. reesei*, which might directly or indirectly act to regulate the main transcription factors controlling cellulase gene expression in this fungus.

### The gene expression of Sugars, Amino Acids, and Ions transporters is Regulated by the MAPK Signaling Pathway in *T. reesei*

The RNA-Seq analysis identified distinct transporters that were differentially expressed in Δ*tmk1* and Δ*tmk2* in the presence of sugarcane bagasse. Efficient nutrient transport is important for proper fungal development. In the *T. reesei* genome, approximately 5% (459) of all genes encode proteins with transport function. Among these genes, 6% (30 genes) exhibited altered expression in Δ*tmk1*, while expression of 7% (35 genes) was altered in Δ*tmk2* when compared to QM6a in the presence of sugarcane bagasse (Supplementary Tables 11 and 12). Our results showed that modulation of transporter expression mediated by MAPK is carbon source-dependent.

In Δ*tmk1*, five genes were upregulated and 17 downregulated exclusively in the presence of sugarcane bagasse (Supplementary Table 11). The largest group of differentially expressed transporters in this condition belonged to the MFS family of permeases. Four MFS permeases were upregulated in Δ*tmk1*, and the gene encoding the most strongly induced MFS permease gene (ID 65915), was expressed at least 3-fold higher in the mutant strain than the parental strain. In addition, 9 genes encoding MFS permeases were downregulated in Δ*tmk1*. The gene with ID 54005 was strongly downregulated in Δ*tmk1*, nearly 70-fold decreased expression when compared to QM6a. Interestingly, one gene encoding copper transporter (IDs 52315) was also repressed in the mutant strain during growth in sugarcane bagasse, indicating the influence of MAPK signaling on cellulose oxidation status. The involvement of copper transporters in the cellulose oxidation status was also suggested by Antonieto *et al*.^37^.

The role of TMK2 in the transporter system of *T. reesei* during sugarcane bagasse degradation was also analyzed. In the presence of sugarcane bagasse, 25 genes were upregulated and 10 were downregulated. In this condition, most differentially regulated genes belonged to the MFS permease family. From the 25 upregulated genes, 21 encoded MFS permeases (Supplementary Table 12). The most strongly induced gene encoded a maltose permease (ID 65191), which was expressed 38-fold higher in the mutant strain. A MFS permease-coding gene (ID 54005) was the most strongly repressed gene in Δ*tmk2*, with 32-fold lower expression in the mutant when compared to the parental strain.

**Figure 7:**
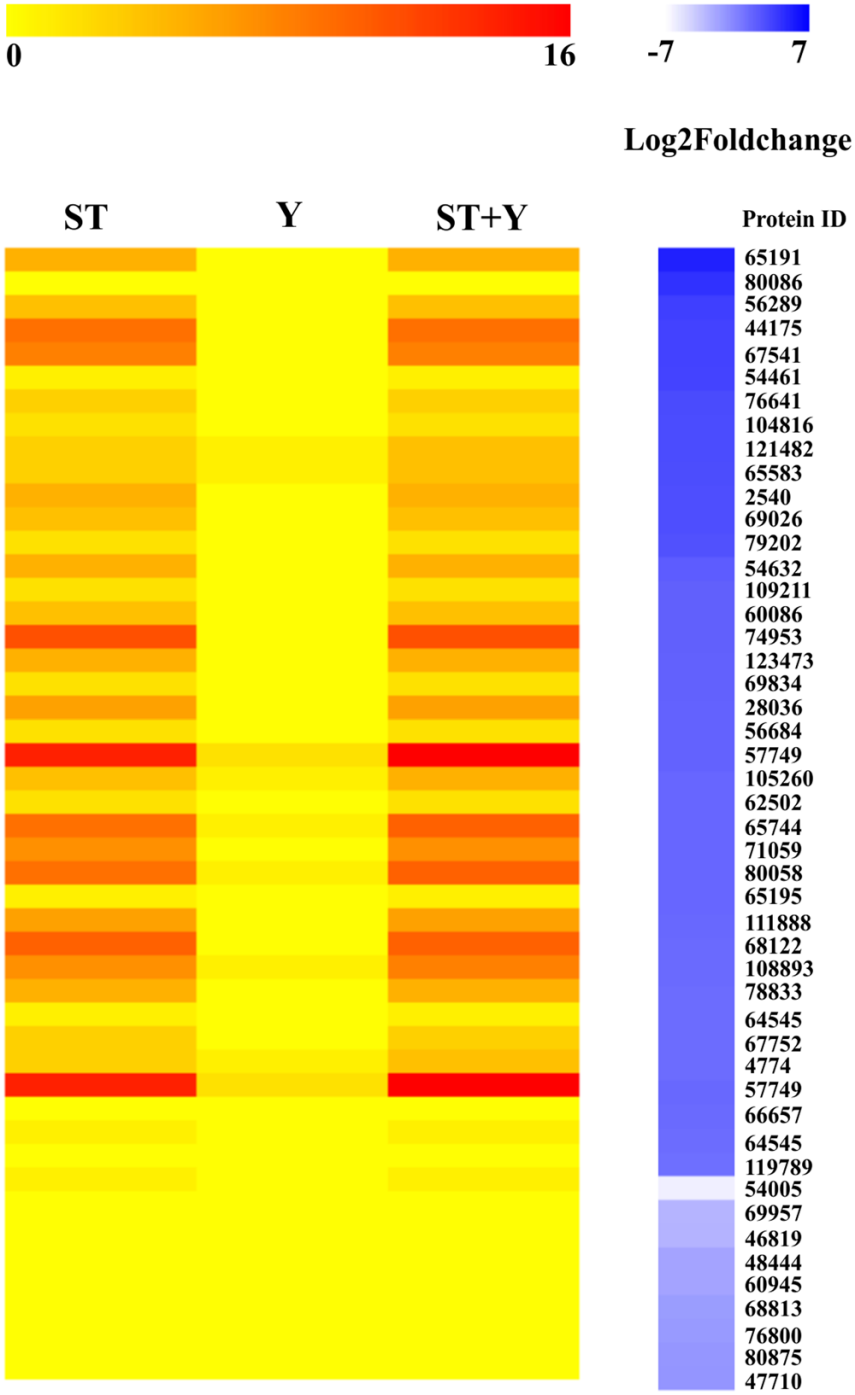
Heat map of predicted MAPK phosphorylation sites of MFS transporters in the Δ*tmk1* and Δ*tmk2* strains grown in sugarcane bagasse. ST: serine and threonine residues are phosphorylated; Y: phosphorylation occurs in tyrosine residues and ST + Y: phosphorylation sites in all three residues, serine, threonine, and tyrosine.

Interestingly, *in silico* identification of MAPK phosphorylation sites suggested that MFS transporters might be regulated by post-translational modifications such as phosphorylation. Figure 7 shows, the ST (Serine-Threonine), Y (Tyrosine), and ST + Y (Serine-Threonine-Tyrosine) phosphorylation profiles of MFS genes differentially expressed in Δ*tmk1* and Δ*tmk2* grown in sugarcane bagasse, when compared to the parental strain. The phosphorylation profiles were correlated with the Log2 fold change values of the MFS genes, and ST and ST + Y were the most conserved phosphorylation profiles among the MFS transporters (Fig. 7). Interestingly, we identified a pattern in the expression profile using Log2 fold change values, where the genes with multiple phosphorylation sites were upregulated in the mutant strains in the presence of sugarcane bagasse, while the genes with only a few MAPK phosphorylation sites were the most repressed in the mutant strains (Fig. 7).

## Discussion

Here, we described the gene regulatory network of *T. reesei* Δ*tmk1* and Δ*tmk2* grown in the presence of sugarcane bagasse, glucose, or glycerol. The RNA-Seq data showed that deletion of *tmk1* and *tmk2* affects different processes in *T. reesei*. In the Δ*tmk1*, we observed the downregulation of the main cellulase genes in *T. reesei* and the differential expression of genes mostly encoding MFS family transporters. Our results showed that the MAPK genes examined might regulate a range of processes in *T. reesei*, being some similar processes probably regulated by different mechanisms. The growth of Δ*tmk2* in the presence sugarcane bagasse and glucose promoted the regulation of cellulase transcription as well as the modulation of genes related to carbohydrate, amino acid, and lipid metabolism, and secondary metabolism. We demonstrated the differential expression of genes related to different signaling pathways such as GPCRs, phospholipases, calcium and MAPK signaling, as well as transporter families like the MFS permeases, and sugar transporters.

Regarding cellulolytic genes, different reports have demonstrated that cellulase expression is regulated at the transcriptional level and in a carbon source-dependent manner^2,32^. However, the molecular mechanisms that control these processes remain poorly understood^33^. The involvement of MAPKs in cellulase gene expression was recently suggested. First, Wang *et al*.^24^ showed that TMK3 positively regulates both cellulase transcription and production in *T. reesei*. Additionally, it was demonstrated that there are MAPK phosphorylation sites in the main transcription factors involved in cellulase expression such as XYR1, CRE1, ACE1, and ACE2, suggesting that the MAPK pathway might regulate the activity of these transcription factors that in turn regulate cellulase expression. However, our data did not show the direct modulation of these transcription factors by TMK1 and TMK2, suggesting indirect regulation by other transcription factors in *T. reesei*^34^. TMK2 has been suggested to be involved in the control of cell wall integrity, sporulation, and cellulase production^28^. Deletion of *ime2*, encoding a MAPK in *T. reesei* induced the expression of the three main cellulase genes (*cbh1, cbh2*, and *egl1*) and repressed the expression of XYR1 and CRE1^29^. Recently, it was shown that deletion of the MAPK *tmk1* leads to improved growth and significantly improved cellulase formation in the presence of wheat bran and Avicel^30^. Our global transcriptional network analysis showed that deletion of *tmk1* and *tmk2* profoundly affected cellulase expression in *T. reesei*, but did not affect XYR1 or CRE1. Interestingly, this regulatory mechanism seems to occur in an unusual way, with TMK2 acting as both a positive and negative regulator of cellulase expression, whereas TMK1 was a positive regulator of cellulase expression (Supplementary Tables 13 and 14). Furthermore, our results showed that different CAZymes were differentially expressed between the Δ*tmk1* and Δ*tmk2*, suggesting a specific pattern of cellulase expression regulation in this organism.

In Δ*tmk1* grown in the presence sugarcane bagasse, we observed repression of the main cellulase genes of *T. reesei*, including *bgl1, egl2/cel5a, cbh1/cel7a, egl1/cel7b*, and *cbh2/cel6;* the main xylanases *xyn3, xyn2* and *xyn4*; the mannanase *man1*; lytic polysaccharide monooxygenases *cel61b* and *cel61a*; the carbohydrate esterase *axe1*; and the arabinofuranosidases *abf2* and *abf3*. On the other hand, in the presence of glucose, genes encoding exoglucanases, mannosidases, and galactosidases were de-repressed. This result suggests that TMK1 might be involved in carbon catabolite repression (CCR). This regulation profile was totally different in Δ*tmk2*, in which polysaccharides lyases, glycosyl transferases, chitinases, one xylanase (*xyn5*), and one β-glucosidase (*bgl2/cel1a*) were the most upregulated holocellulases upon growth with sugarcane bagasse, while genes encoding glycosyl hydrolases, glycosyl transferases, carbohydrate-binding module enzymes, and carbohydrate esterases were mainly downregulated in this condition. Enzyme activity assays showed that CMCase and β-glucosidase activities were increased in Δ*tmk2* grown in the presence of sugarcane bagasse, while these enzyme activities were decreased in Δ*tmk1* when compared to the parental strain. These results are in accordance to our transcriptomic data that pointed to an induction of different cellulolytic genes in Δ*tmk2*. For Δ*tmk1*, decreased CMCase activity might be explained by the downregulation of the main CAZy genes when the mutant strain was grown in the presence of sugarcane bagasse. Similarly, Wang *et al*.^28^ demonstrated increased CMCase and β-glucosidase activities when the mutant strain was grown in wheat bran and cellulose. This decrease in β-glucosidase activity was also observed in the Δ*tmk3* mutant strain grown in cellulose^24^. In Δ*tmk1*, decreased xylanase activity might be explained by the downregulation of the main xylanase genes when the mutant strain was grown in the presence of sugarcane bagasse. The involvement of MAPK signaling in the cellulase activity has previously been shown in *T. reesei*^24,28,30,35^, however, here we showed for the first time that the deletion of a member of MAPK cascade can also regulate xylanase activity in this fungus. Regarding β-xylosidase activity, we observed a decrease in enzyme activity in both mutant strains in the presence of sugarcane bagasse. Curiously, β-xylosidase gene expression was not differentially regulated in either mutant strain when compared to the QM6a parental strain. This effect was also observed upon the deletion of MAPK TMK3^24^. These results suggested that a yet-unknown mechanism might regulate β-xylosidase production and/or induction by MAPK signaling control. Together, our results suggest that TMK2 may play a dual role in regulating cellulase expression, acting as a positive and negative regulator of this process in *T. reesei*, probably depending on the carbon source.

The involvement of MAPK signaling in regulating cellulase expression was previously demonstrated in *Cochliobolus heterostrophus*, a pathogenic plant fungus, in which deletion of MAPK gene *chk1* modulated the expression of cellobiohydrolase 7 (CBH7) and endoglucanase 6 (EG6) during plant infection^36^. Accordingly, our data showed that the expression of the main transcription factors involved in the control of cellulase expression did not change in Δ*tmk1* and Δ*tmk2* grown in the presence glucose, glycerol, or sugarcane bagasse. Likewise, transcriptional data of Δ*xyr1* and Δ*cre1* strains in the presence of cellulose, sophorose, and glucose showed that none of the transcription factors responsible for cellulase expression were differentially expressed in comparison to the expression in the parental strain^37–39^. Curiously, among the 92 differentially expressed transcription factor-encoding genes in Δ*tmk2* strain grown in the presence of sugarcane bagasse, glucose, or glycerol, 25 genes were common to transcription factors differentially expressed in the Δ*xyr1* strain in the presence of cellulose, sophorose, and glucose^38^. Seven other differentially expressed transcription factors in Δ*tmk2* were also equally modulated in Δ*cre1* grown on cellulose and sophorose^37,39^. These findings suggest an indirect relationship between MAPK signaling and XYR1 and CRE1 activation, that must be elucidated.

Amongst the differentially regulated transcription factors, in Δ*tmk2* in the presence of sugarcane bagasse, three genes (IDs 4124, 1941, and 34221) encode Myb family transcription factors, which control a range of cellular processes, such as proliferation, apoptosis, differentiation, metabolism, and stress response^40–42^. In *A. nidulans* and *Fusarium graminearum*, Myb transcription factors are important regulators of nitrogen metabolism^43,44^. The most repressed gene (ID 72524) in Δ*tmk2* in the presence of sugarcane bagasse encodes a bZIP transcription factor that is also involved in nitrogen metabolism in *A. nidulans*^45^. Similarly, four other bZIP members (IDs 65315, 110152, 103372, and 110152) showed 3-fold, 2-fold, and 7-fold lower expression in the mutant strain grown on sugarcane bagasse, glucose, and glycerol, respectively. Therefore, our results suggested that the MAPK signaling pathway might regulate *T. reesei* nitrogen metabolism and growth in the carbon sources studied. In Δ*tmk1*, only eighteen transcription factors were differentially expressed in the presence of sugarcane bagasse, glucose, and glycerol. The most strongly induced gene in this mutant in the presence of sugarcane bagasse and glucose compared to the parental strain encode Zn2Cys6 and C2H2 transcriptional regulator family members. The transcriptional regulator PacG/VIB-1 (ID 54675), required for the completion of meiosis and sporulation, was the unique downregulated transcription factor in Δ*tmk1* grown in sugarcane bagasse. Curiously, this gene was also downregulated in Δ*tmk2* in the same condition, suggesting a possible mechanism of cross-talk between the two MAPK genes in *T. reesei*.

The transcriptome data of Δ*tmk2* revealed that the MAPK signaling pathway regulates the expression of different genes encoding other signaling pathways during growth in the presence of sugarcane bagasse. In this strain, genes encoding GPCRs, phospholipases, Ras GTPases, and kinase proteins were overexpressed in the presence of sugarcane bagasse. In contrast, genes related to GPCRs, calcium, histidine kinases, and upstream components of MAPK signaling, such as MAPKKK Ste11 and its regulator Ste50, showed decreased expression in this condition. In *Cryphonectria parasitica*, G-protein signaling controls the expression of the cellobiohydrolase *cbh1* in the presence of cellulose^46^. Similarly, in *T. reesei*, the G proteins GNA3, GNB1, and GNG1 positively regulate cellulase expression in the presence of light^10,47^. The cross-talk between G proteins and MAPK has been described in *S. pombe*, in which the pheromone response is regulated by the MAPK Spk1, the protein kinase Ras1, and Gα-GTP, being the latter responsible for activating downstream components of this signaling pathway^48,49^. Regarding GPCRs, we demonstrated that eight PTH11-related genes were the most common GPCRs expressed during the growth of Δ*tmk2* in sugarcane bagasse. These GPCRs are a new class of receptors described in *Magnaporthe grisea* involved in pathogenic response^50,51^. In *F. graminearum*, the differential expression of PTH11 members seems to be related to fungus-host adaptation^52^. In *Neurospora crassa*, genes encoding PTH11 members were differentially expressed upon growth in cellulose, suggesting that these proteins might play a yet-unclear key role in plant cell wall recognition and in the control of holocellulolytic enzyme expression^53^. Therefore, our results suggested a recognition mechanism for different carbon sources involving PTH11 GPCRs in the activation of MAPK TMK2 during growth of *T. reesei* in the presence of sugarcane bagasse. Interestingly, it has been demonstrated in the cellulolytic bacterium *Ruminococcus flavefaciens* that the phosphorylation and dephosphorylation dynamics are important for carbon source recognition and regulation of carbon metabolism^54^. Similarly, a nutrient-specific mechanism involving G proteins and MAPK signaling was suggested to explain the ability of the fungus *Cochliobolus heterostrophus* to recognize different carbon sources and survive in diverse environments^55^. Together, our results pointed to a dual mechanism of carbon source recognition where the differentially expressed genes are components upstream to TMK2 and TMK1 that might be responsible for sensing the initial carbon source and in turn might initiate the degradation signal for these different substrates in *T. reesei*. However, more studies are necessary to elucidate this mechanism.

Regarding nutrient transport, MFS transporters were the most-modulated classes of transporters in the Δ*tmk1* and Δ*tmk2* mutant strains. The MFS permeases represent a large family of secondary transporters carrying small solutes, such as monosaccharides, oligosaccharides, amino acids, peptides, vitamins, and enzyme cofactors^56–58^. In yeast, transporters belonging to the MFS family have been found to play key roles in sugar uptake^59–61^. In *A. nidulans*, two MFS transporters are involved in transporting lactose, xylose, glucose, galactose, and mannose, indicating they accept multiple sugars as a substrate^62,63^. The lack of specificity of theses transporters could be explained by the similar structure of different sugars, and some transporters can act both as transporters and nutrient sensors. In *T. reesei*, MFS transporters are highly expressed during growth in cellulose and lactose^64^. Similarly, the transcriptome profiling of Δ*xyr1* and Δ*cre1* revealed that MFS permeases are highly repressed in the presence of cellulose^37–39^. Moreover, in *T. reesei*, the MFS protein Ctr1 plays a significant role in cellulolytic signaling, acting as a sophorose transporter^65^. Another report showed that Ctr1 is upregulated in the presence of lactose, probably acting as a lactose permease^66^. Our results showed that in Δ*tmk1*, two MFS permease-encoding genes (IDs 48444 and 69957) were specifically downregulated in the presence of sugarcane bagasse. These transporters are highly similar to a putative maltose permease of the human pathogenic fungus *Talaromyces marneffeii* and may be involved in disaccharide transport^67^. The maltose permease gene (ID 69957) was also downregulated in the Δ*xyr1* grown on cellulose^38^. Recently, Nogueira *et al*.^68^ showed that the novel *T. reesei* 69957-sugar transport system (*Tr69957*) is capable of transporting xylose, mannose, and cellobiose. The deletion of *Tr69957* in *T. reesei* affected the fungal growth and biomass accumulation, and the sugar uptake in the presence of mannose, cellobiose, and xylose. Additionally, the MFS permease with ID 46819, which was repressed in Δ*tmk1* in the presence of sugarcane bagasse, is homologous to the putative cellodextrin transporter-like protein CLP1 of *N. crassa*, which is involved in cellulase induction through a mechanism involving repression of a cellodextrin transporter, in turn inhibiting cellodextrin uptake^69^. Together, our results suggest that MAPK signaling may regulate the expression of different transporter families in *T. reesei*, and these transporters seem to play a promiscuous role in signaling pathways involved in cellulase production.

## Conclusions

Our study contributes to a better understanding of the role of the MAPK intracellular signaling pathway in the degradation of cellulosic biomass by *T. reesei*. Transcriptomic analysis showed that several genes exhibited altered expression in a carbon source-dependent manner. The transcript profiles of Δ*tmk1* and Δ*tmk2* were drastically altered during growth in the presence of sugarcane bagasse. The most differentially expressed genes included mainly cellulolytic enzymes, genes encoding proteins involved in signaling pathways such as GPCRs and calcium signaling, as well as transporters and transcription factors. We highlighted the modulation of different classes of transporters that seem to be involved in a refined mechanism of sugar recognition. Interestingly, our results revealed a duality of MAPK signaling pathway in the control of metabolic processes and cellulase expression, in which TMK2 seems to be more responsible controlling basal development of *T. reesei*, while TMK1 showed a specialist role in the regulation of cellulase expression in this fungus.

## Methods

### Fungal Strain and Growth Conditions

*Trichoderma reesei* strain QM6aΔ*tmus53*Δ*pyr4*^31^ was used in this study. This strain was obtained from the Institute of Chemical Engineering & Technical Biosciences of Vienna University of Technology, TU Vienna, Austria. The strain was maintained at 4 °C on MEX medium (malt extract 3% (w/v) and agar-agar 2% (w/v)), which was supplemented with 5 mM uridine in the case of the *pyr4* deletion strain.

*T. reesei QM6aΔtmus53Δpyr4* parental, Δ*tmk2* and *Δtmk1* mutant strains were grown on MEX medium at 28 °C for 7–10 days until complete sporulation. For gene expression assays, a spore suspension containing approximately 10^7^ cells mL^−1^ was inoculated into 200 mL of Mandels-Andreotti medium^10^ containing 1% (w/v) sugarcane bagasse, 2% (w/v) glucose, or 1% glycerol as the sole carbon source. The exploded sugarcane bagasse was prepared as previously described^70^. Briefly, sugarcane bagasse *in natura* was treated with 14 kg/cm^2^ water steam, washed exhaustively with distilled water until reducing sugars were not detected by 3,5-dinitrosalicylic acid (DNS)^71^, and dried at 40 °C for several days. Sugarcane bagasse was kindly donated by Nardini Agroindustrial Ltd., Vista Alegre do Alto, São Paulo, Brazil. The cultures were incubated on an orbital shaker (200 rpm) at 28 °C for 48 hours for the sugarcane bagasse and glucose experiments. For the experiments with sugarcane bagasse, the parental, *Δtmk2* and Δ*tmk1* strains were grown in glycerol 1% (w/v) for 24 hours and then transferred to a medium containing sugarcane bagasse. All experiments were performed in three biological replicates. The resulting mycelia were collected by filtration, frozen in liquid nitrogen, and stored at −80 °C for RNA extraction. For phenotypic assays, the parental and mutant strains were inoculated on minimal medium plates containing 1% of one of the following carbon sources: glucose, starch, sucrose, lactose, glycerol, cellulose (Avicel) MEX, as well as on potato dextrose agar plates^24,28^.

### Vector Construction for Gene Deletion

Deletions of *tmk2* (Tr_82351) and *tmk1* (Tr_121539) of *T. reesei* were generated as previously described^72^. To construct the deletion cassette, the orotidine-5′-phosphate decarboxylase gene of *T. reesei* (*pyr4*, Tr_74020) was used as a selection marker. Sequences were obtained from the *T. reesei* genome database (http://genome.jgi.doe.gov/Trire2/Trire2.home.html). The marker gene and its respective 5′ and 3′ flanking sequences were amplified using the primers described in Additional file 2. The primers were designed and analyzed with the OligoAnalyzer Tool (https://eu.idtdna.com/calc/analyzer). To enable yeast-mediated recombination of the deletion cassette, the external 5′-UTR forward (F) and 3′-UTR reverse (R) primers possessed cohesive ends with the vector pRS426^73,74^ and the internal primers 5′-UTR R and 3′-UTR F contained cohesive ends with 5′ and 3′sequences of *pyr4* gene (Additional file 2). The 50 μL reaction mixture contained 1 U Platinum Taq DNA Polymerase High Fidelity (Thermo Scientific), 1 × High Fidelity PCR Buffer, 0.2 mM dNTPs, 0.3 µM forward and reverse primers, 1 µL *T. reesei* QM9414 genomic DNA (150 ng/µL) as a template, and nuclease-free water. The PCR fragments were purified using a QIAquick PCR Purification Kit (Qiagen).

For yeast-mediated recombination, the yeast shuttle vector pRS426 (*amp*^R^ *lacZ* URA3)^73,74^ was used, which was digested with *Eco*RI and *Xho*I (Thermo Scientific) and was purified with the QIAquick PCR Purification Kit (Qiagen). Yeast transformation was essentially conducted as previously described^75–77^. An overnight culture (200 rpm, 30 °C) of the yeast *S. cerevisiae* strain SC9721 (*MATα his3-Δ200 URA3-52 leu2Δ1 lys2Δ202 trp1Δ63*) (Fungal Genetic Stock Center – www.fgsc.net) was prepared. Then, 1 mL of the overnight culture was added to 50 mL of fresh YPD (1% yeast extract, 2% peptone, 1% glucose) (all from Sigma Aldrich) medium and incubated at 30 °C until OD_600_ = 1. Thereafter, the cells were centrifuged and resuspended in 100 mM lithium acetate for transformation. To this end, equimolar quantities of both 5′ and 3′ flanking regions, *pyr4*, and digested pRS426 were mixed and used for yeast transformation through the lithium acetate method^78^. *S. cerevisiae* SC9721 transformants were selected for their ability to grow on YPD medium supplemented with lysine, histidine, leucine, and tryptophan, without uracil. *S. cerevisiae* genomic DNA was extracted by as previously described^79,80^. The deletion cassettes of Tr_82351 and Tr_121539 were PCR-amplified using TaKaRa Ex Taq DNA Polymerase (Clontech) using 5F and 3R primers (Additional file 2).

### Transformation of *T. reesei*

*T. reesei* QM6aΔ*tmus53*Δ*pyr4* was used for transformation to achieve highly efficient homologous integration of the deletion cassette. Protoplast transformation was carried out as previously described^81^. For transformation, 10 μg of linear purified deletion cassette fragment was used. Transformants were grown on selective minimal medium [1 g/L MgSO_4_•7H_2_O, 10 g/L 1% KH_2_PO_4_, 6 g/L (NH_4_)_2_SO_4_, 3 g/L trisodium citrate•2H_2_O, 10 g/L glucose, 20 mL/L 50X trace elements solution (0.25 g/L FeSO_4_•7H_2_O, 0.07 g/L ZnSO_4_•2H_2_O, 0.1 g/L CoCl_2_•6H_2_O, 0.085 g/L MnSO_4_•H_2_O), 2% (wt/vol) noble agar lacking uridine] (all from Sigma Aldrich). Cassette integration was verified by PCR using the primer *pyr4*Sc (inside in the selectable marker gene *pyr4*) and the gene-specific primer 82351Sc or 121539Sc (outside the transformation cassette) (Additional file 2). The expression profiles of the genes Tr_82351 and Tr_121539 were also analyzed by RT-qPCR.

### RT-qPCR Analysis

For expression analysis, total RNA (1 µg) from each sample was digested with DNAse I (Fermentas) to remove genomic DNA. cDNA was synthesized using the Maxima™ First Strand cDNA Synthesis kit (Fermentas) according to the manufacturer’s instructions. The cDNA was diluted 1:50 and used as a template for real-time PCR. Reactions were run in the Bio-Rad CFX96™ by using SsoFast™ EvaGreen® Supermix (Bio-Rad) for detection according to the manufacturer’s instructions. Each reaction (10 µL) contained 5 µL of SsoFast™ EvaGreen® Supermix (Bio-Rad), forward and reverse primers (500 nm each; Additional file 2), cDNA template, and nuclease-free water. PCR cycling conditions were as follows: 10 min at 95 °C, followed by 40 cycles of 10 s at 95 °C and 30 s at 60 °C, and a ramp of 60–95 °C a rate of 0.5 °C/10 s to generate a melting curve to test for primer dimers and nonspecific amplification. β-Actin transcript was used as an internal reference to normalize the amount of total RNA present in each reaction^82^. For sugarcane bagasse expression analysis, gene expression levels of genes were calculated from the threshold cycle according to the 2^−ΔΔCT^ method^83^, relative to transcript levels in the parental strain QM6a grown in non-induced condition (glycerol) for 24 h^82^. For glucose, gene expression levels were calculated from the threshold cycle according to the 2^−ΔCT^ method^83^, relative to transcript levels of β-Actin. Finally, *tmk1* and *tmk2* expression levels in the mutant strains were calculated from the threshold cycle according to the 2^−ΔΔCT^ method^83^, relative to transcript levels in QM6a.

### Southern Blotting

The selected transformants were analyzed by Southern hybridization as previously described^84^, to demonstrate homologous integration of the transformation cassettes at the targeted *T. reesei QM6aΔtmus53Δpyr4* loci. For this analysis, 25 µg of total genomic DNA of both parental and mutant strains were digested overnight with *Eco*RI and *Hind*III (Δ*tmk2*) and *Pst*I and *Eco*RV (Δ*tmk1*) (Fermentas) and then, digested DNA was transferred to GE Healthcare Amersham Hybond -N+ Membranes (GE). The probe was produced from a PCR-amplified fragment using 82351-5 F and 82351-5 R (promoter region of *tmk2*), and 121539-3 F and 121539-3 R (terminator region of *tmk1*) primer sets (Additional file 2) and was labeled by using a digoxigenin DNA labeling kit (Roche, Mannheim, Germany), following the manufacturer’s instructions. Labeling, hybridization, and immunological detection were carried out with a nonradioactive labeling and immunological detection kit with CDP-Star as the chemiluminescent substrate (Roche, Mannheim, Germany), as previously described^85^.

### Enzyme Activity Assays

CMCase activity was analyzed in microplates (96-well PCR plate) following the protocol described by Xiao *et al*.^86^ with some modifications: 30 μL of 1% carboxymethylcellulose (CMC), previously prepared in sodium acetate buffer at pH 4.8, was added to each well with 30 μL of enzyme. After a 30-min incubation at 50 °C, 60 μL of DNS were added, followed by heating at 95 °C for 5 min to allow color development. Then, the samples were transferred to a flat-bottomed microplate and absorbance at 540 nm was read. One enzyme unit was defined as the amount of enzyme capable of releasing 1 μmol of reducing sugar per minute. β-glucosidase and β-xylosidase activities were respectively assayed by abilities to hydrolyze pNPG and pNPX following published protocols^2,87,88^. Xylanase activity was determined by mixing 25 µL of enzyme solution with 50 µL of xylan from beechwood (1.0 mg mL^−1^) in 100 mM sodium acetate buffer (pH 5.0) for 30 min at 50 °C. After incubation, 75 µL of DNS were added and heated at 95 °C for 5 min for color development. After this, 100 µL of the solution were transferred to a microliter plate, and the absorbance at 540 nm was read. One unit of enzyme was defined as the amount of enzyme capable of releasing 1 µmol of reducing sugar per minute^32^.

### RNA Extraction, Library Construction, and Sequencing

Total RNA was extracted from mycelia of each sample using a Direct-zol RNA MiniPrep (Zymo Research), according to the manufacturer’s instructions. RNA concentrations were determined by a fluorometric Qubit RNA HS Assay Kit (Thermo Scientific) and RNA integrity was verified by the Agilent 2100 Bioanalyzer. Total RNA of three biological replicates of parental, Δ*tmk2*, and Δ*tmk1* strains grown in the presence of sugarcane bagasse (48 hours), glucose (48 hours), and glycerol (24 hours), resulting in 27 samples was used for library preparation, using the Illumina TruSeq Stranded mRNA Sample Preparation Kit (Illumina), according to the manufacturer’s instructions. The libraries were quantified using a KAPA Library Quantification Kit Illumina (KAPA Biosystems) and were sequenced at NGS sequencing facility at Laboratório Nacional de Ciência e Tecnologia do Bioetanol (CTBE) (Campinas, São Paulo) on an Illumina Hiseq2500 platform with paired-end 2 × 100-bp reads. RNA-Seq data from the 27 libraries were deposited in the Gene Expression Omnibus (GEO) under accession number GSE100602.

### RNA-Seq Data Analysis

In the current study, Illumina Hiseq2500 technology was used for sequencing, resulting in approximately 143 million, 139 million, and 158 million 2 × 100-bp paired-end reads for parental, Δ*tmk2*, and Δ*tmk1* strains, respectively. Quality-filtered reads (Additional file 3) were mapped to the *T. reesei* 2.0 reference genome, available at JGI Genome Portal (http://genome.jgi-psf.org/Trire2/Trire2.home.html), using the TopHat2 v2.0.4 aligner^89^, allowing two mismatches and only unique alignments. After alignment, Samtools version 0.1.18^90^ was used to process the alignment data, which were visualized using the Integrative Genomics Viewer^91^. Genes were annotated using the *T. reesei* 2.0 reference genome, a local database provided by Prof. C. P. Kubicek (TU, Vienna), and the InterPro database (http://www.ebi.ac.uk/interpro/)^92,93^. The R package DESeq2 version 1.6.3^94^ was used to perform the differential expression analysis using the raw number of reads mapped to each gene in each sample to perform statistical tests, based on the negative binomial distribution. To analyze the expression pattern, we used the following strategy: first, we used the libraries of glycerol to compare the data of parental and mutant strains grown on sugarcane bagasse (QM6a sugarcane bagasse/QM6a glycerol, Δ*tmk2* glycerol/QM6a glycerol, Δ*tmk2* sugarcane bagasse/Δ*tmk2* glycerol, Δ*tmk1* glycerol/QM6a glycerol, and Δ*tmk1* sugarcane bagasse/Δ*tmk1* glycerol). Then, the data of the parental and mutant strains grown on sugarcane bagasse were compared to each other (Δ*tmk2* sugarcane bagasse/QM6a sugarcane bagasse and Δ*tmk1* sugarcane bagasse/QM6a sugarcane bagasse). For glucose, the data of the mutant strain were directly compared to those of the parental strain (Δ*tmk2* glucose/QM6a glucose and Δ*tmk1* glucose/QM6a glucose). The DESeq2 package was used for normalization, using the median log deviation, and for the differential expression analysis, we applied an adjusted p-value ≤ 0.05 as the threshold. Thus, Log 2-fold change values ≥ 1 and ≤−1 represent up- and downregulation. All statistical analyses were performed using the R package^95^.

### Functional Enrichment and Network Analysis

Differentially expressed transcripts were annotated using Blast2GO^96^. For functional enrichment analysis, differentially expressed genes were annotated to GO terms using the topGO algorithm^97^. Significantly enriched GO terms (p ≤ 0.05), were further analyzed using GraphPad Prism v 5.00 Software. To reconstruct the regulatory network of Δ*tmk2*/QM6a and Δ*tmk1*/QM6a under the experimental conditions analyzed, differentially expressed genes in Δ*tmk2* (2215 in sugarcane bagasse, 1991 in glucose, and 1754 in glycerol in total) and in Δ*tmk1* (258 in sugarcane bagasse, 803 in glucose, and 32 in glycerol in total) were analyzed using the Cytoscape 3.0.1 software^98^, following a previously reported procedure^38^.

## Electronic supplementary material

Supplementary information

Dataset 1

Dataset 2

Dataset 3

Dataset 4

Dataset 5

Dataset 6

Dataset 7

Dataset 8

Dataset 9

Dataset 10

Dataset 11

Dataset 12

Dataset 13

Dataset 14
