# Supplementary material for: The Duality of the MAPK Signaling Pathway in the Control of Metabolic Processes and Cellulase Production in Trichoderma reesei
Source: Sci Rep. 2018 Oct 8;8:14931. doi: 10.1038/s41598-018-33383-1 (PMC6175961; doi:10.1038/s41598-018-33383-1)
Supplement: Supplementary file 1 — Supplementary information [file 41598_2018_33383_MOESM1_ESM.pdf]

Supplementary information

The Duality of the MAPK Signaling Pathway in the Control of Metabolic Processes and Cellulase Production in *Trichoderma reesei*

Renato Graciano de Paula[1], Amanda Cristina Campos Antoniêto[1], Cláudia Batista Carraro[1], Douglas Christian Borges Lopes[1], Gabriela Felix Persinoti[2], Nalu Teixeira Aguiar Peres[3], Nilce Maria Martinez-Rossi[4], Rafael Silva-Rocha[5], and Roberto Nascimento Silva[1]*

[1]Molecular Biotechnology Laboratory, Department of Biochemistry and Immunology, Ribeirao Preto Medical School (FMRP), University of Sao Paulo, Ribeirao Preto, SP, Brazil, renatocbufes@yahoo.com.br, amandaantonieto@yahoo.com.br and claudiacarraro@usp.com, douglas.chr.94@gmail.com, [2]Laboratório Nacional de Ciência e Tecnologia do Bioetanol (CTBE), Centro Nacional de Pesquisa em Energia e Materiais (CNPEM), Campinas, SP, Brazil, gabriela.persinoti@bioetanol.org.br, [3]Center of Biological Science and Health (CCBS), Sergipe Federal University (UFS), Aracaju, SE, Brazil, naluperes@gmail.com, [4]Department of Genetics, Ribeirão Preto Medical School, University of São Paulo, Ribeirão Preto, SP, Brazil, nmmrossi@usp.br, [5]Systems and Synthetic Biology Laboratory, Department of Cell and Molecular Biology, Ribeirao Preto Medical School (FMRP), University of Sao Paulo, Ribeirao Preto, SP, Brazil, silvarochar@gmail.com

*Correspondence to: Roberto do Nascimento Silva
Department of Biochemistry and Immunology
Ribeirao Preto Medical School, University of Sao Paulo
Ribeirao Preto 14049-900, SP, Brazil, Tel.: +55 16 3602 3112,
Fax: +55 16 3602-0219, E-mail: rsilva@fmrp.usp.br.

1. **Supplementary figures and Supplementary Figure Legends**

<u>Supplementary figure 1</u>

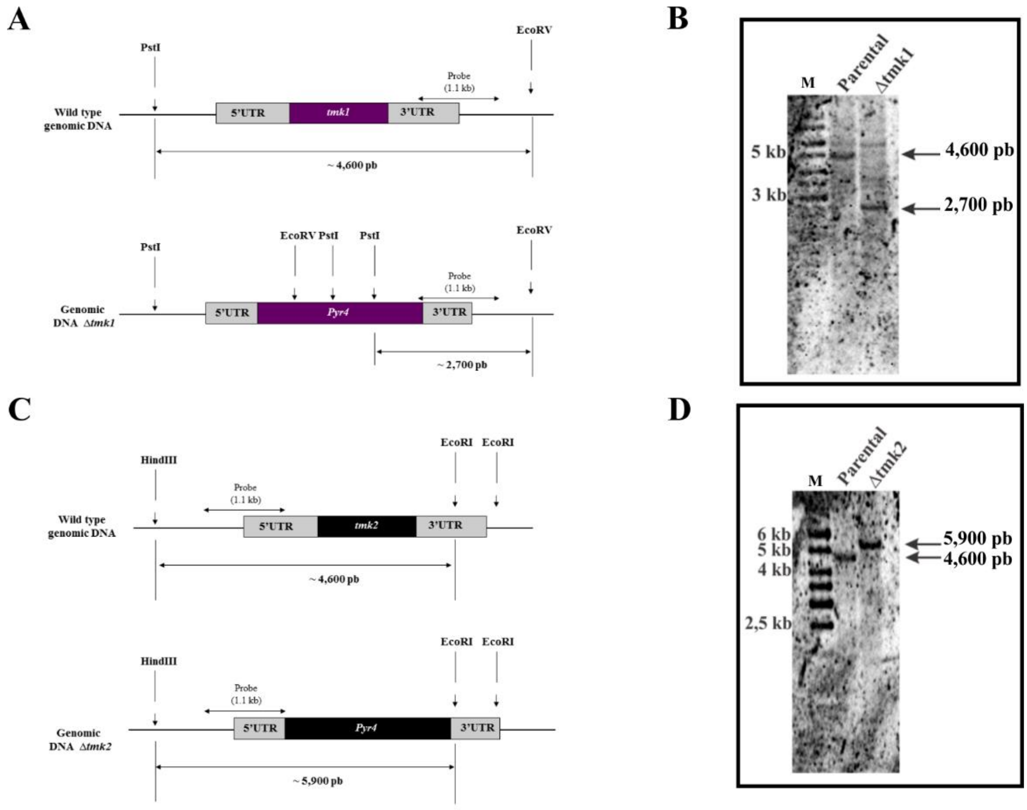

Figure S1. Southern blotting analysis of Δ*tmk2*, Δ*tmk1*, and QM6a strains. (A) Schematic representation of the enzymatic restriction pattern of *tmk1* deletion. **(B)** Southern blotting analysis of *T. reesei* parental and Δ*tmk1* strains confirming the deletion of *tmk1*. **(C)** Schematic representation of the enzymatic restriction pattern of *tmk2* deletion. **(D)** Southern blotting analysis of *T. reesei* parental and Δ*tmk2* strains confirming the deletion of *tmk2*. M, DNA molecular size marker. Predicted sizes of DNA fragments hybridized with the probe are indicated by arrows.

Supplementary figure 2

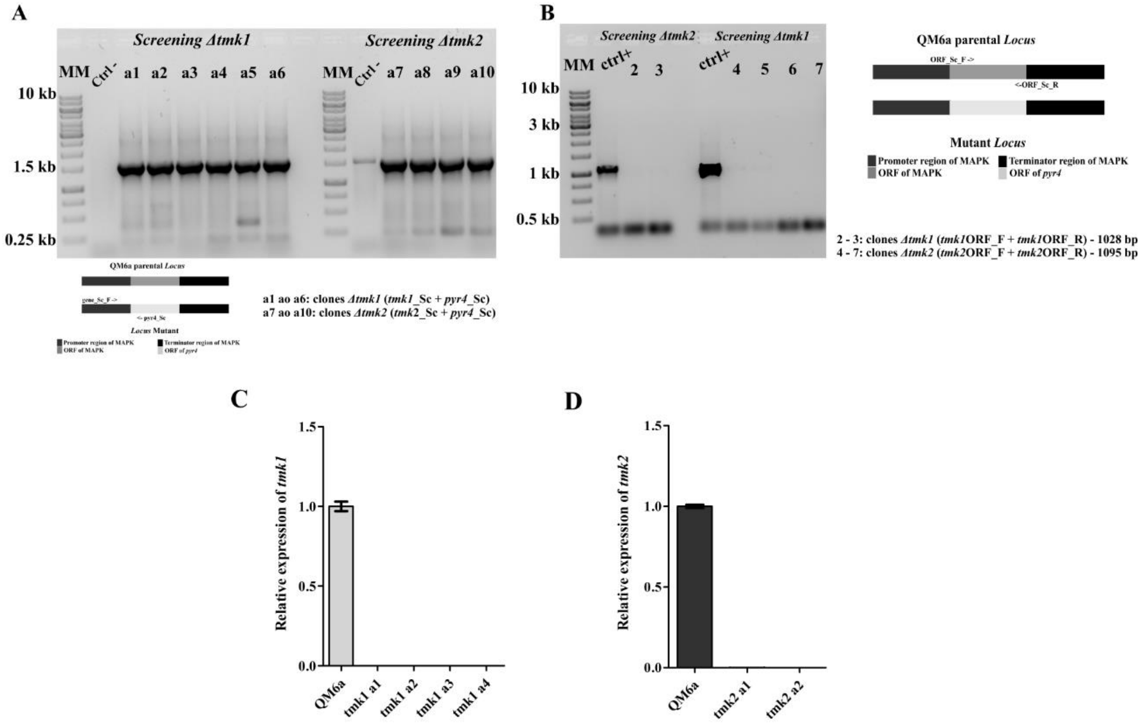

Figure S2. PCR and qRT-PCR for mutant strains identification. (A) PCR for amplification of molecular marker (*pyr4*) for validation of Δ*tmk1* and Δ*tmk2* mutant strains. Ctrl$^-$: negative control (as template was used DNA of QM6a parental strain), **(B)** PCR for amplification of coding region of *tmk1* and *tmk2* for validation of Δ*tmk1* and Δ*tmk2* mutant strains. Ctrl+: positive control (as template was used DNA of QM6a parental strain). MM, DNA molecular size marker, **(C)** qRT-PCR expression analysis of *tmk1* for validation of Δ*tmk1* mutant strain. cDNA of QM6a parental strain was used as positive control, **(D)** qRT-PCR expression analysis of *tmk2* for validation of Δ*tmk2* mutant strain. cDNA of QM6a parental strain was used as positive control.

Supplementary figure 3

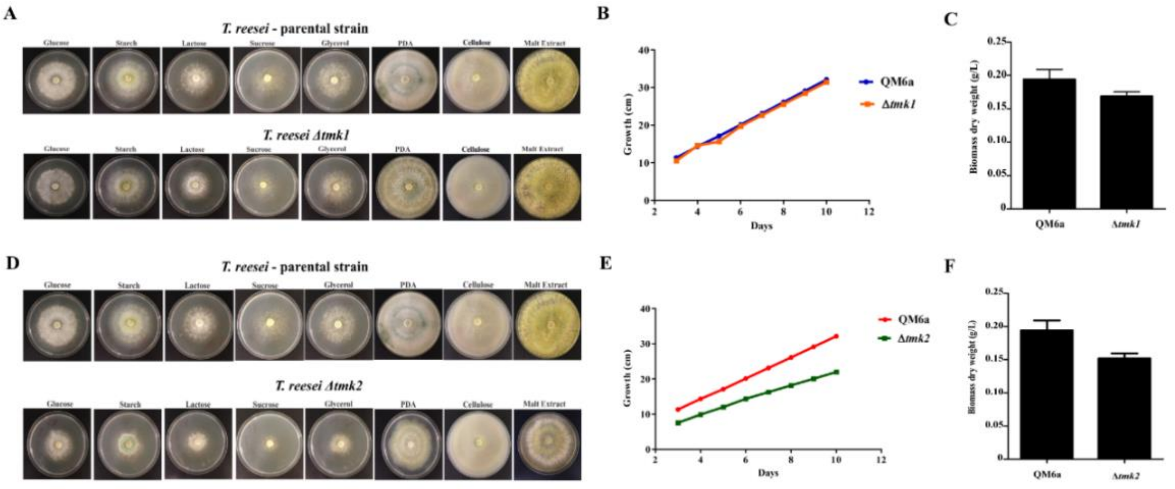

Figure S3. Phenotypic characterization of Δ*tmk1* and Δ*tmk2* mutant strains. (A) Growth of the *T. reesei* parent strain and Δ*tmk1* on plates with different carbon sources. The growth of mutant and parental strains was analyzed after four days of cultivation. **(B)** Race tubes growth analysis. The growth of mutant and parental strains was analyzed through ten days of cultivation. **(C)** Growth of *T. reesei* QM6a and Δ*tmk1* strains in glycerol as carbon source. Error bars represent data from three biological replicates. No significant differences in growth were observed between the Δ*tmk1* mutant strain and the parental QM6a. **(D)** Growth of the *T. reesei* parental strain and Δ*tmk2* on plates with different carbon sources. The growth of mutant and parental strains was analyzed after four days of cultivation. **(E)** Race tubes growth analysis. The growth of mutant and parental strains was analyzed throughout ten days of cultivation. **(F)** Growth of the *T. reesei* parental strain QM6a and Δ*tmk2* in glycerol as a carbon source. Error bars represent data from three biological replicates. No significant differences in growth were observed between the Δ*tmk2* mutant strain and the parental QM6a.

Supplementary figure 4

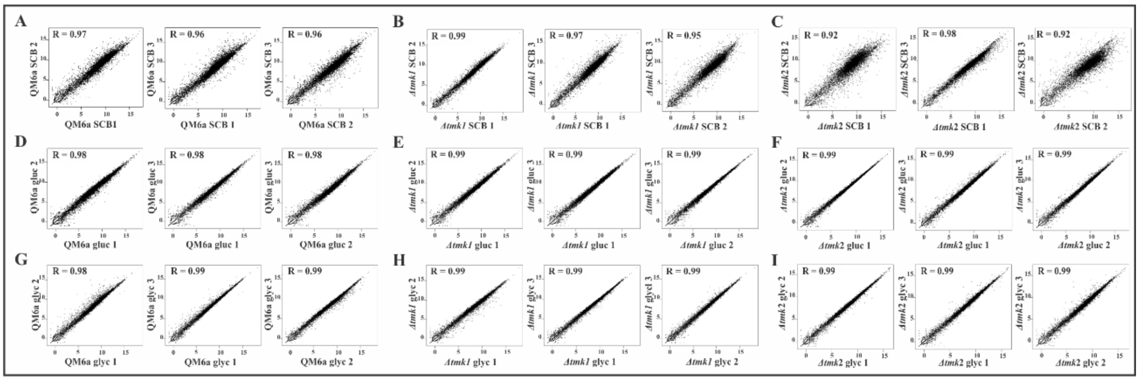

Figure S4. **Biological replicates used for the RNA-Seq analysis (Δ*tmk1*, Δ*tmk2,* and QM6a). (A)** Biological replicates of QM6a grown in sugarcane bagasse (SCB). Graphs are representing the Pearson correlation between biological replicates of each sample demonstrating the reliability of RNA-Seq (R ≥ 0.96). **(B)** Biological replicates of Δ*tmk1* grown in sugarcane bagasse (SCB). Graphs are representing the Pearson correlation between biological replicates of each sample demonstrating the reliability of RNA-Seq (R ≥ 0.95). **(C)** Biological replicates of Δ*tmk2* grown in sugarcane bagasse (SCB). Graphs are representing the Pearson correlation between biological replicates of each sample demonstrating the reliability of RNA-Seq (R ≥ 0.92). **(D)** Biological replicates of QM6a grown in glucose (gluc). Graphs representing the Pearson correlation between biological replicates of each sample demonstrating the reliability of RNA-Seq (R ≥ 0.98). **(E)** Biological replicates of Δ*tmk1* grown in glucose (gluc). Graphs are representing the Pearson correlation between biological replicates of each sample demonstrating the reliability of RNA-Seq (R ≥ 0.99). **(F)** Biological replicates of Δ*tmk2* grown in glucose (gluc). Graphs are representing the Pearson correlation between biological replicates of each sample demonstrating the reliability of RNA-Seq (R ≥ 0.99). **(G)** Biological replicates of QM6a grown in glycerol (glyc). Graphs representing the Pearson correlation between biological replicates of each sample demonstrating the reliability of RNA-Seq (R ≥ 0.998). **(H)** Biological replicates of Δ*tmk1* grown in glycerol (glyc). Graphs are representing the Pearson correlation between biological replicates of each sample demonstrating the reliability of RNA-Seq (R ≥ 0.99). **(I)** Biological replicates of Δ*tmk2* grown in glycerol (glyc). Graphs are representing the Pearson correlation between biological replicates of each sample demonstrating the reliability of RNA-Seq (R ≥ 0.99).

Supplementary figure 5

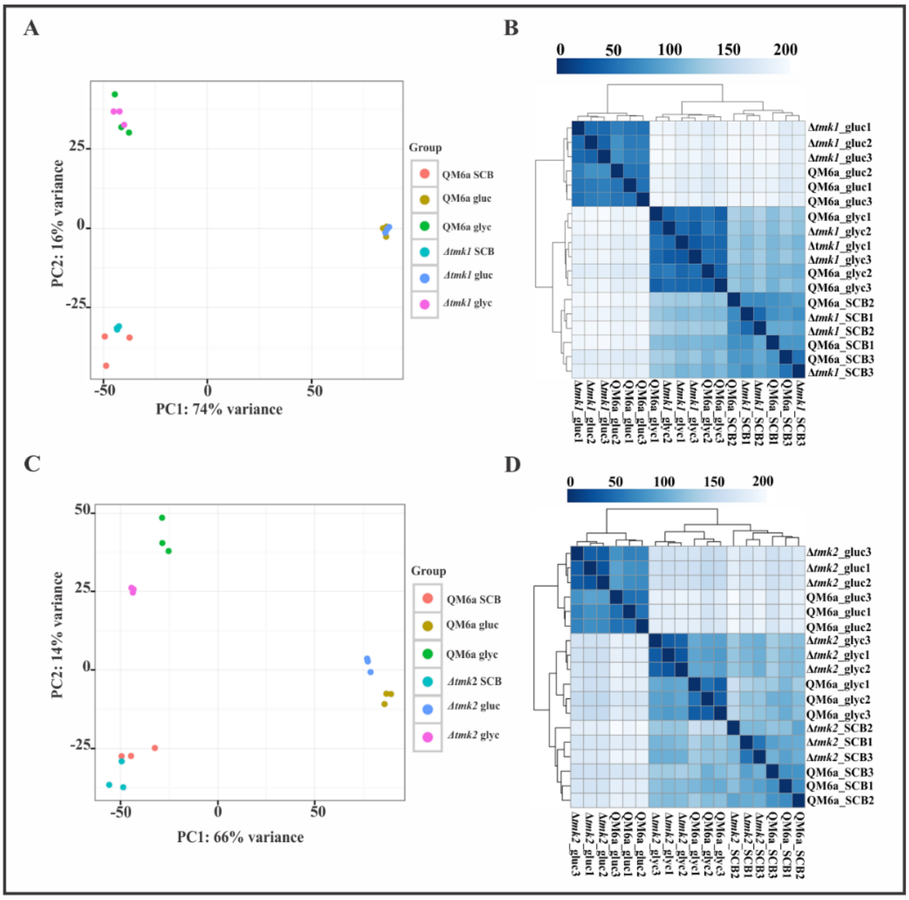

Figure S5. Principal component analysis (PCA) and hierarchical clustering of sample-to-sample distance of Δ*tmk1*, Δ*tmk2*, and QM6a strains analyzed in this study. (A) Principal component analysis of the QM6a and Δ*tmk1* samples analyzed to assess the overall similarity between the conditions using DESeq2's plotPCA. **(B)** Hierarchical clustering of the QM6a and Δ*tmk1* samples analyzed to assess the overall similarity between the conditions. **(C)** Principal component analysis of the QM6a and Δ*tmk2* samples to assess the overall similarity between the conditions using DESeq2's plotPCA. **(D)** Hierarchical clustering of the QM6a and Δ*tmk2* samples analyzed to assess the overall similarity between the conditions. SCB: sugarcane bagasse, GLUC: glucose, and GLY: glycerol.

Supplementary figure 6

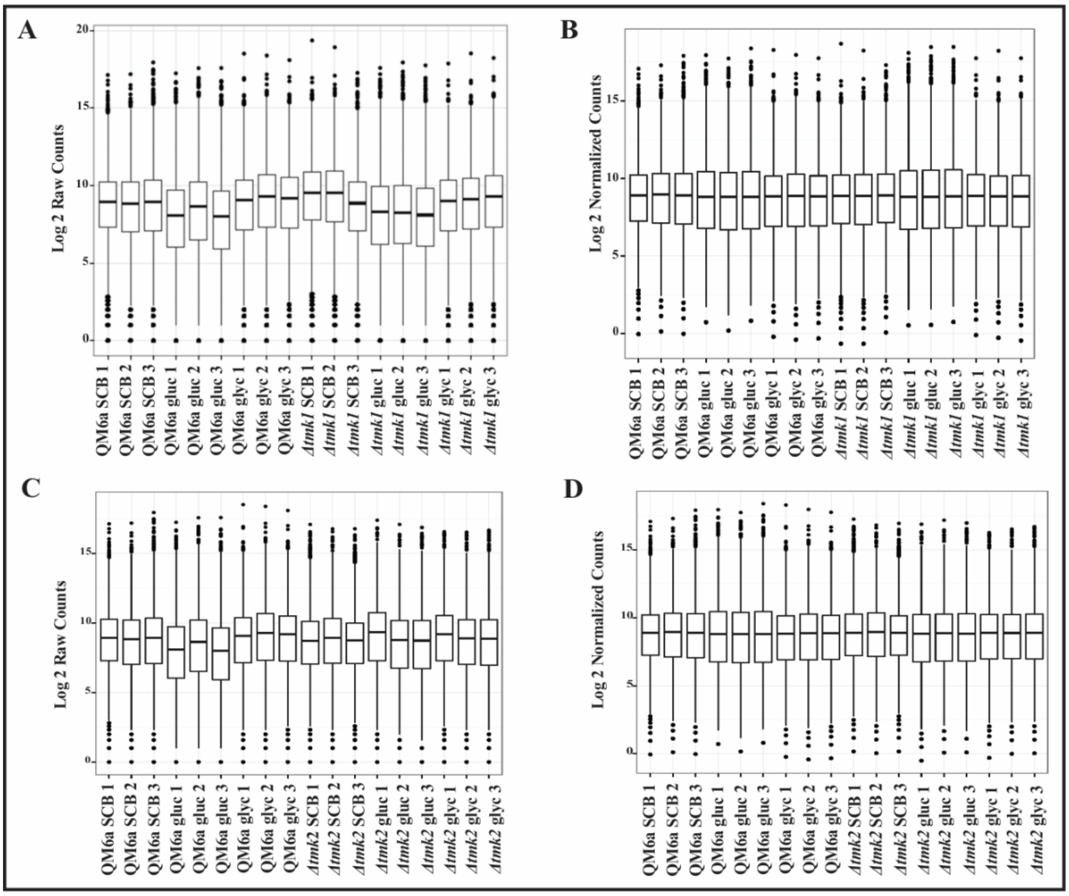

Figure S6. Box plot of all samples pre- and post-normalization. (A) Raw counts of QM6a and Δ*tmk1*, **(B)** Normalized counts of QM6a and Δ*tmk1*, (**C**) Raw counts of QM6a and Δ*tmk2*, and **(D)** Normalized counts of QM6a and Δ*tmk2*.

Supplementary figure 7

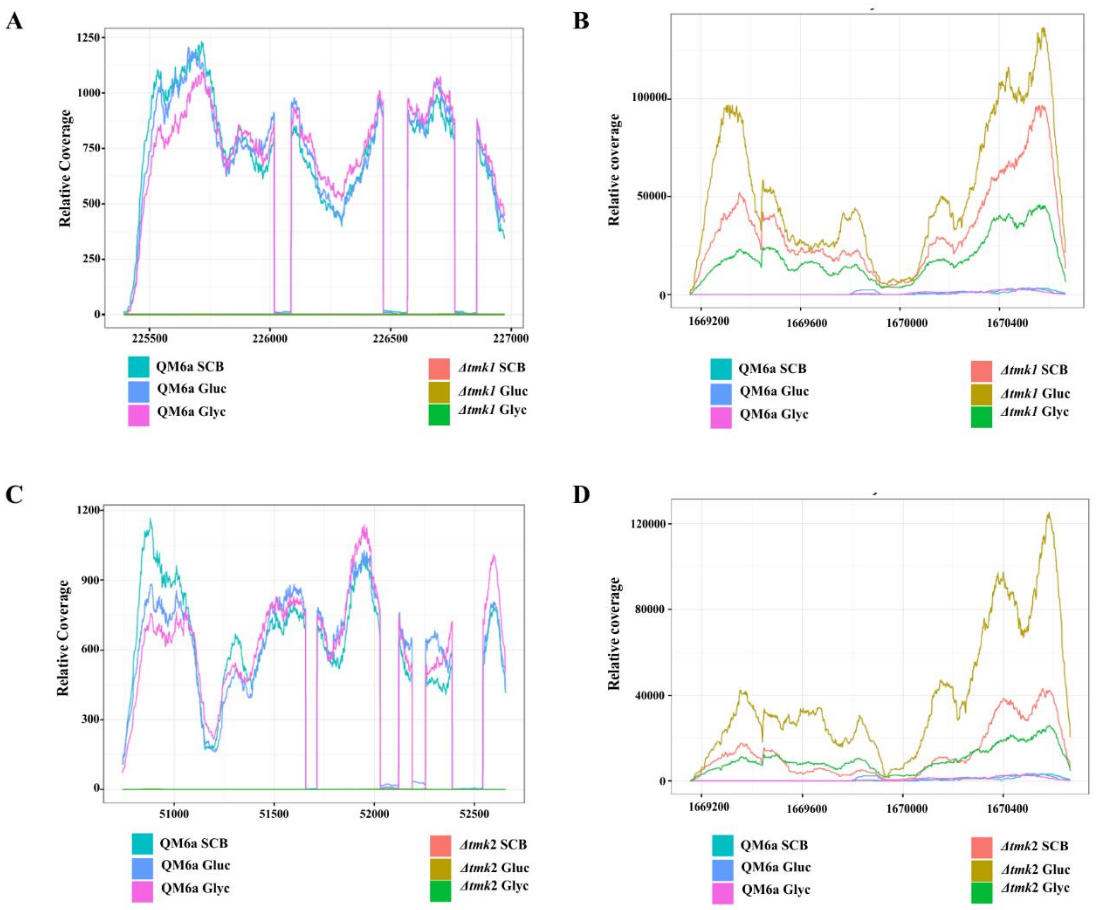

Figure S7. RNA-Seq coverage plots of the *tmk1* and *tmk2* genes. (A) Number of sequence reads (x-axis) (Reads per Million) is shown along the genomic region corresponding to the *tmk1* gene (Protein ID 121539, scaffold 8, genomic position 112698-114278). **(B)** Number of sequence reads (x-axis) (Reads per Million) is shown along the genomic region corresponding to the *pyr4* gene in the Δ*tmk1* strain (Protein ID 74020, scaffold 1, genomic position 1669155-1670667). **(C)** Number of sequence reads (x-axis) (Reads per Million) is shown along the genomic region corresponding to the *tmk2* gene (Protein ID 82351, scaffold 36, genomic position 50743-52656). **(D)** Number of sequence reads (x-axis) (Reads per Million) is shown along the genomic region corresponding to the *pyr4* gene in the Δ*tmk2* strain (Protein ID 74020, scaffold 1, genomic position 1669155-1670667). SCB: sugarcane bagasse, GLUC: glucose, and GLY: glycerol.

Supplementary figure 8

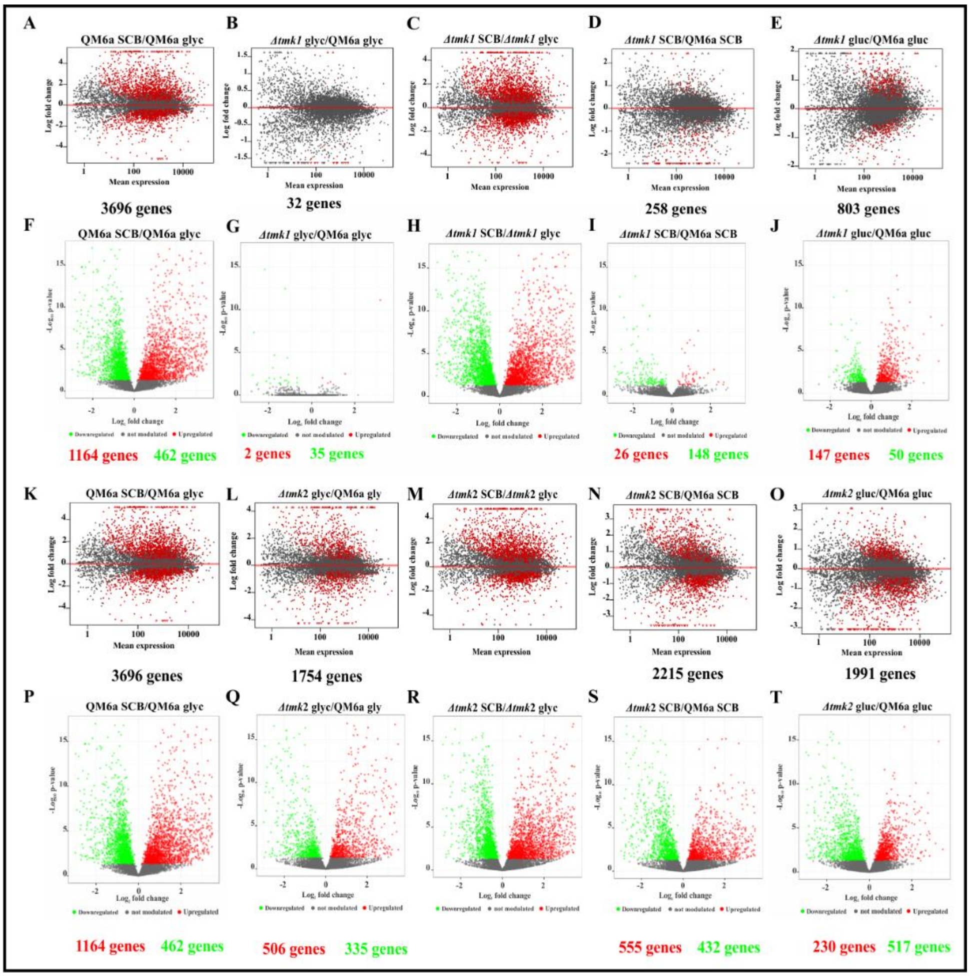

Figure S8. Differentially expressed genes (DEG) in Δ*tmk1* and Δ*tmk2* compared to parental strain QM6a grown in glucose, glycerol, and sugarcane bagasse as sole carbon sources. (A) QM6a SCB/QM6a glyc, **(B)** Δ*tmk1* glyc/QM6a glyc, **(C)** Δ*tmk1* SCB/Δ*tmk1*glyc, **(D)** Δ*tmk1* SCB/QM6a SCB, **(E)** Δ*tmk1* gluc/QM6a gluc. Differentially expressed genes identified by the DESeq2 package are plotted in red (padj ≤ 0.05). **(F-J)** Volcano plot of differentially expressed genes in Δ*tmk1* under all conditions studied. Log 2-fold changes and their corresponding −log 10 p-values of all genes in the RNA-Seq are shown. Upregulated genes (log 2-fold change ≥1 and padj ≤ 0.05) are depicted in red and downregulated (log2 fold change ≤ −1 and padj ≤ 0.05) in green. **(K)** QM6a SCB/QM6a glyc, **(L)** Δ*tmk2* glyc/QM6a glyc, **(M)** Δ*tmk2* SCB/ Δ*tmk2* glyc, **(N)** Δ*tmk2* SCB/QM6a SCB, **(O)** Δ*tmk2* gluc/QM6a gluc. Differentially expressed genes identified by the DESeq2 package are plotted in red (p ≤ 0.05). **(P-T)** Volcano plot of differentially expressed genes in Δ*tmk2* under all conditions studied. Log2 fold changes and their corresponding −log 10 p-values of all genes in RNA-Seq are shown. Upregulated genes (log2 fold change ≥1 and padj ≤ 0.05) are depicted in red and downregulated (log2 fold change ≤ −1 and padj ≤ 0.05) in green. SCB: sugarcane bagasse, GLUC: glucose, and GLY: glycerol.

Supplementary figure 9

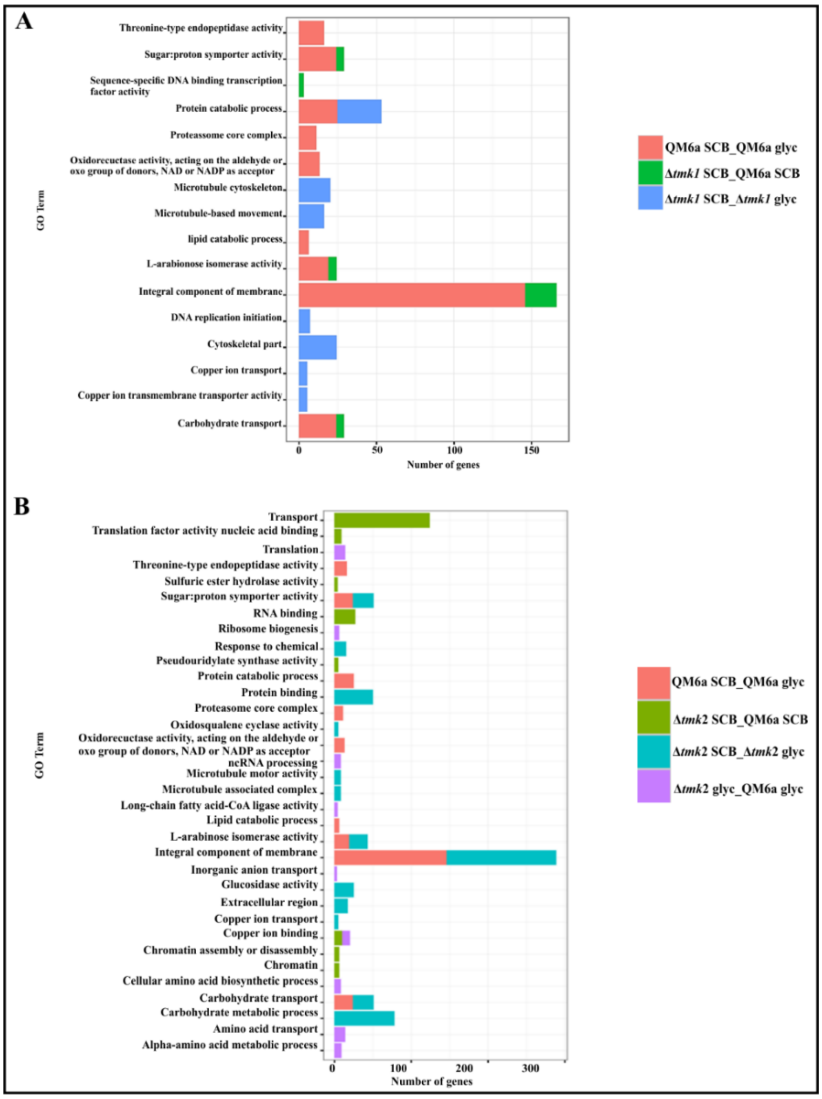

Figure S9. Functional categorization of differentially expressed genes in the Δ*tmk1*, Δ*tmk2*, and QM6a strains. (A) Enrichment analysis of differentially expressed genes in the Δ*tmk1* mutant strain. **(B)** Enrichment analysis of differentially expressed genes in the Δ*tmk2* mutant strain. The enriched GO terms according to molecular, cellular component, and biological process in *T. reesei*. Significantly enriched categories (p ≤0.05) are shown. The threshold for calling differentially expressed genes was (padj ≤0.05). SCB: sugarcane bagasse, GLUC: glucose, and GLY: glycerol.

2. Supplementary files

Additional file 1. Deletion of *tmk2* and *tmk1* in *T. reesei* and phenotypic characterization

T. reesei Δ*tmk2* and Δ*tmk1* strains were constructed by homologous recombination using *QM6aΔtmus53Δpyr4* as the parental strain, whose non-homologous end joining pathway was disrupted [31]. Southern blot analysis of *Eco*RV- and *Pst*I-digested chromosomal DNA (Supplementary Figure 1A) using the terminator region of *tmk1* gene (Tr_121539) as a probe resulted in expected signals in both parental (4,600 bp) and Δ*tmk1* (2,700 bp) mutant strains, respectively (Supplementary Figure 1B). Integration of the deletion cassette was confirmed by PCR using the primers *pyr4*_Sc and 121539_Sc (Additional file 2). In this case, integration at the homologous locus resulted in a specific amplicon with a size about 1,500 bp (Supplementary Figure 2A) depending on the exact location of the outside primer. In addition, no amplification was observed in the mutant strain Δ*tmk1* when a pair of primers that annealed in the coding region of *tmk1* is used (Supplementary Figure 2B). Moreover, the mRNA of *tmk1* was not detectable in the mutant strain (Supplementary Figure 2C). Regarding Δ*tmk2*, analysis of *Eco*RI- and *Hind*III-digested chromosomal DNA (Supplementary Figure 1C) using the promoter region of the *tmk2* gene 82351 as a probe resulted in expected signals in the parental strain (4,600 bp) and in the Δ*tmk2* mutant strain (5,900 bp) (Supplementary Figure 1D). Integration of the deletion cassette was also evaluated by PCR using *pyr4*_Sc and 82351_Sc primers (Additional file 2). In this case, integration at the homologous locus resulted in a specific amplicon of approximately 1500 bp (Supplementary Figure 2A). Additionally, no amplification was observed in the mutant strain Δ*tmk2* when a pair of primers that annealed in the coding region of *tmk2* is used. An amplicon of 1,000 bp was detected only in the parental strain (Supplementary Figure 2B). Moreover, *tmk2* mRNA

was not detected in the mutant strain (Supplementary Figure 2D). These results confirmed the complete deletion of *tmk1* and *tmk2* genes in the fungal genome.

Phenotypic analysis was conducted in parental, *Δtmk1,* and *Δtmk2* strains by growing these strains on agar plates with different media compositions (Supplementary Figures 3A, 3D). The *Δtmk1* mutant strain showed no changes in growth and sporulation patterns compared with the parental strain. A slight reduction of growth on agar plates was observed for the *Δtmk1* mutant strain when the mutant strain was grown in minimal media containing lactose as a carbon source (Supplementary Figure 3A). In addition, we observed that both the parental and mutant strains had a similar growth profile in race tube experiments and biomass formation (Supplementary Figures 3B, 3C). Generally, sporulation patterns of the *Δtmk2* mutant strain also did not differ from the parental strain. However, a noticeable reduction of growth on agar plates was observed for the *Δtmk2* mutant strain when grown in minimal media supplemented with lactose and glucose (Supplementary Figure 3D). In addition, we observed that both the parental and mutant strains had distinct growth profiles in race tube experiments (Supplementary Figure 3E), while the parental and *Δtmk2* mutant strains showed similar biomass accumulation after growth on glycerol at 24 hours (Supplementary Figure 3F). Taken together, the results suggested that only *tmk2* is involved in regulating vegetative growth.

Additional file 2. Primer sequences used in this study.

Primer	Sequence	Amplicon	Amplification of
Pyr4-F	5'-ATTCCAACGCCTCTTCTTTGTGCTTTTCT-3'	3222 bp	Selectable marker
Pyr4-R	5'-ATTCCGACATATGGAAGCTGATATCGTCG-3'		
121539_5F	5'-**GTAACGCCAGGGTTTTCCCAGTCACGACG**TTGATCCCTCTGTGTCAACC-3'	1161 bp	5'-flanking region of TR121539
121539-5R	5'-CGACGATATCAGCTTCCATATTCCGACTAGAACTGAAGAGGAGTTGAGG-3'		
121539_3F	5'-AGAAAAGCACAAAGAAGAGGCTCCAACTAGTAGGTGTGGTCAGTGATGG-3'	1009 bp	3'-flanking region of TR121539
121539_3R	5'-**GCGGATAACAATTTCACACAGGAAACAG**CTGTAGCAGTCTCTCTTGTCG-3'		
Pyr4_Sc	5'-CCTCTTTTTCCATCTTGTC-3'	1580 bp	Screening of *T. reesei* transformants
121539_Sc	5'-ATGGCCTTTCATATTGCTG-3'		
121539_RT_F	5'-GGACAAAAGGTTGCCATCAAG-3'	130 bp	qPCR-RT
121539_RT_R	5'-CTTCTGGATGTCAAGGATGGAG-3'		
121539ORF_F	5'-GCAAGATCTCCTTCAACGTCA-3'	1028 bp	ORF amplification
121539ORF_R	5'- CCGCATAATCTCCTGGTAAAT-3'		
82351_5F	5'-**GTAACGCCAGGGTTTTCCCAGTCACGACG**CACCTCTGTATCTCAAGACG-3'	1134 bp	5'-flanking region of TR82351
82351-5R	5'-CGACGATATCAGCTTCCATATTCCGACTATATGTTGTCTGAGTCCCAGC-3'		
82351_3F	5'-AGAAAAGCACAAAGAAGAGGCTCCAACTAGTTATCTGAGTGGTGTGTGG-3'	1000 bp	3'-flanking region of TR82351
82351_3R	5'-**GCGGATAACAATTTCACACAGGAAACAG**CCCATGTTCTACGACATCAGC-3'		
Pyr4_Sc	5'-CCTCTTTTTCCATCTTGTC-3'	1580 bp	Screening of *T. reesei* transformants
82351_Sc	5'- ATCTCATGTAGCTCGGTCA -3'		
82351_RT_F	5'- CTTCCAGTCCTTCATCTACCAG -3'	132 bp	qPCR-RT
82351_RT_R	5'- GCCAAAGTCGCAAATCTTCAG -3'		
82351ORF_F	5'- CTTCAACCAGGACTTTGTGGT -3'	1095 bp	ORF amplification
82351ORF_R	5'- AGCGGGACTTGAATCTGCTGG -3'		

*bps given in bold represent sequences overlapping with vector pRS426 for yeast mediated recombination.
**underlined bps represent sequences overlapping with selection marker cassette for yeast mediated recombination.

Additional file 3. Analysis of RNA Sequencing Data

T. reesei QM6a, Δ*tmk1*, and *Δtmk2* strains were cultivated in sugarcane bagasse, glycerol, and glucose as unique carbon sources, and three biological replicates of each condition were submitted for RNA sequencing using an Illumina HiSeq™ 2500. Approximately 143 million of 2x100 bp paired-end reads were obtained for QM6a, 139 million for the mutant strain Δ*tmk2*, and 158 million for the Δ*tmk1* with concordant pair alignment rates of 97.3, 97.6, and 98%, respectively. Regarding the number of nucleotides, the data obtained corresponded to 28.63 GB for QM6a, 27.87 GB, for Δ*tmk2* and 31.74 GB for Δ*tmk1* (Supplementary Table 1). Before expression analysis, data quality assessment was performed using DESeq2 package and a high Pearson correlation was obtained upon comparing the corresponding replicates of QM6a ($R \geq 0.96$) (Supplementary Figures 4A, 4D, 4G), Δ*tmk1* ($R \geq 0.99$) (Supplementary Figures 4B, 4E, 4H), and Δ*tmk2* ($R \geq 0.92$) (Supplementary Figures 4C, 4F, 4I) for the three studied conditions. For Δ*tmk1* and Δ*tmk2,* a principal component analysis (PCA) (Supplementary Figures 5A, 5C), a hierarchical clustering of sample-to-sample distance (Supplementary Figures 5B, 5D), and box plot graphics (Supplementary Figure 6) were performed to ensure the reliability of the data and to demonstrate that the samples were comparable.

To confirm the deletion of the *tmk1* and *tmk2* genes in the respectively mutant strains, the files generated after read alignment were visualized using IGV and the number of reads mapping to *tmk1* and *tmk2* was analyzed. The absence of coverage in *tmk1* and *tmk2* genes was observed in the respective mutant strains for all conditions examined, confirming the presence of the mutation and eliminating any possibility of contamination during strain growth (Supplementary Figures 7A, 7C, respectively). As expected, many reads mapping to *tmk1* and *tmk2* were observed in the QM6a strain during growth in all conditions tested. Our data also showed that there was no expression of the *pyr4* gene (selection marker) in

the QM6a parental strain (Supplementary Figures 7B, 7D, respectively). In contrast, the expression of *pyr4* was observed in the mutant strains.

Here, we described for the first time the effects of deleting the MAPK genes *tmk2* and *tmk1* in *T. reesei*, through the transcriptome analysis of this fungus grown in three different carbon sources. As observed in Supplementary Figures 8A-E and 8K-O, respectively, the expression profile of this filamentous fungus was drastically altered upon the deletion of *tmk1* and *tmk2*. In the QM6a parental strain, we identified 3696 genes (Supplementary Figures 8A, 8K) that were differentially expressed in the presence of sugarcane bagasse, representing 1164 genes upregulated and 462 downregulated in this condition (Supplementary Figures 8F, 8P).

For the Δ*tmk1* mutant strain, we identified 258 genes that were differentially expressed when the mutant strain was grown in the presence of sugarcane bagasse (Supplementary Figure 8D), 803 in glucose (Supplementary Figure 8E), and 32 in glycerol (Supplementary Figure 8B), adopting p-adjusted ≤0.05 as a significance threshold. Among these, a total of 175 genes were upregulated (p-adjusted ≤ 0.05 and Log2 Fold Change ≥1), with 26 in sugarcane bagasse (Supplementary Figure 8I), 147 in glucose (Supplementary Figure 8J), and 2 in glycerol (Supplementary Figure 8G). On the other hand, of the 233 genes that were downregulated (p-adjusted ≤ 0.05 and Log2 Fold Change ≤ −1), 148 were identified in sugarcane bagasse (Supplementary Figure 8I), 50 in glucose (Supplementary Figure 8J), and 35 in glycerol (Supplementary Figure 8G). Of the 9129 genes annotated in the genome of *T. reesei*, 2215 (24.26%) were differentially expressed in the Δ*tmk2* mutant compared to the parental QM6a in the presence of sugarcane bagasse (Supplementary Figure 8N), 1991 (21.8%) in glucose (Supplementary Figure 8O), and 1754 (19.21%) in glycerol (Supplementary Figure 8L), adopting p-adjusted ≤0.05 as a threshold. Among these, a total of 1291 genes were upregulated (p-adjusted ≤ 0.05 and

Log2 Fold Change ≥1), with 555 in sugarcane bagasse (Supplementary Figure 8S), 230 in glucose (Supplementary Figure 8T), and 506 in glycerol (Supplementary Figure 8Q). On the other hand, of the 1284 genes that were downregulated (p-adjusted ≤ 0.05 and Log2 Fold Change ≤ −1), 432 were identified in sugarcane bagasse (Supplementary Figure 8S), 517 in glucose (Supplementary Figure 8T), and 335 in glycerol (Supplementary Figure 8Q).

3. **Supplementary table titles:**

Supplementary Table 1. Summary of RNA-seq reads obtained for the parental and Δ*tmk2* and Δ*tmk1* mutant strains used in this study.

Supplementary Table 2. Genes exclusively regulated in the Δ*tmk1* mutant strain in sugarcane bagasse, glucose and glycerol.

Supplementary Table 3. Genes exclusively regulated in the Δ*tmk2* mutant strain in sugarcane bagasse, glucose and glycerol.

Supplementary Table 4. Top differentially expressed genes in the Δ*tmk1* mutant strain.

Supplementary Table 5. Top differentially expressed genes in the Δ*tmk2* mutant strain.

Supplementary Table 6. Genes differentially expressed in both Δ*tmk1* and Δ*tmk2* strains in sugarcane bagasse as compared to the parental strain.

Supplementary Table 7. Genes differentially expressed in both Δ*tmk1* and Δ*tmk2* strains in the presence of glucose as compared to the parental strain.

Supplementary Table 8. Signaling pathway genes differentially expressed in the mutant strain Δ*tmk2*.

Supplementary Table 9. Transcription factors differentially expressed in the Δ*tmk1* mutant strain.

Supplementary Table 10. Transcription factors differentially expressed in the mutant strain Δ*tmk2*.

Supplementary Table 11. Expression pattern of genes related with transport activity in the Δ*tmk1* mutant strain.

Supplementary Table 12. Expression pattern of genes related with transport activity in the Δ*tmk2* mutant strain.

Supplementary Table 13. CAZymes differential expressed in the Δ*tmk1* mutant strain.

Supplementary Table 14. CAZymes differentially expressed in the *Δtmk2* mutant strain.
